# ﻿Taxonomic novelties in New Guinea Uvarieae (Annonaceae): Revision of *Pyramidanthe* and new genus records of *Desmos* and *Fissistigma*

**DOI:** 10.3897/phytokeys.262.145660

**Published:** 2025-09-12

**Authors:** Zacky Ezedin, Jenifer C. Lopes

**Affiliations:** 1 Harvard University Herbaria, 22 Divinity Ave, Cambridge, Massachusetts 02138, USA Harvard University Herbaria Cambridge United States of America

**Keywords:** Indonesia, *
Mitrella
*, neotype, new species, Papua New Guinea, species complex

## Abstract

The diversity of climbing Annonaceae in New Guinea, the world’s largest tropical island, remains poorly known. Here, we provide a regional taxonomic account for three genera of the Uvarieae tribe, two of which represent new genus records for the island. First, we revise the genus *Pyramidanthe* (formerly *Mitrella*), recognizing a total of seven species. Four are described as new, *P. montana*, *P. nervosa*, *P. oblongifolia*, and *P. parvifolia*, whereas another, *P. silvatica*, is synonymized under *P. schlechteri*. Two additional Uvarieae genera, *Desmos* and *Fissistigma*, are recorded in New Guinea for the first time. *Desmos* is reported with the description of a new species, *D. insolens*, based on a single fruiting collection from the Ramu basin of Papua New Guinea. *Fissistigma*, sister to *Pyramidanthe*, is reported following the discovery of two collections of *F. latifolium* from the Bird’s Head Peninsula of Indonesia. Due to improper typification of the latter species, a neotype is herein designated. A key to the species of *Pyramidanthe* is provided alongside a key to the six climbing Annonaceae genera in New Guinea. This account raises to 21 the total number of Annonaceae genera present on the island.

## ﻿Introduction

Annonaceae Juss. has a pantropical distribution, with 108 genera and ca. 2,500 species (Chatrou et al. 2012; Erkens et al. 2023; Nge et al. 2024). The family is well-studied globally with a stable internal classification based on phylogenetic and phylogenomic data (Chatrou et a. 2012; Nge et al. 2024). Owing to an international network of taxonomic specialists working on the family, our understanding of its diversity and classification is often regarded among the best of tropical families. This can be seen in the relatively high amount of systematic and taxonomic literature available on the Annonaceae in nearly all tropical regions.

The family’s diversity in the Asian tropics encompasses 40 genera and ca. 1,100 species (Turner 2018). Despite this, however, when compared to the Neotropics and Afrotropics, the Annonaceae of the Asia–Pacific remain poorly studied as a whole. This is evidenced by the many new species which continue to be regularly described in the region (e.g., Bunchalee et al. 2022; Randi et al. 2022; Page 2023; Yang et al. 2023; Damthongdee et al. 2024). Recent monographic efforts in the family have been made in certain parts of tropical Asia, namely in Malaysia (Tree Flora of Sabah and Sarawak; Soepadmo et al. 2014) and Thailand (Flora of Thailand; Chayamarit et al. 2022). Additionally, an exhaustive checklist of all currently known species across the Asia–Pacific region is also available (Turner 2018). However, much of the taxonomic research over the past decade has largely focused on mainland Asia, primarily the Indochina region. Meanwhile, the Malesian region has received far less attention, despite containing a greater share of the species diversity.

Of all the islands in Malesia, New Guinea clearly stands out as one of the last major tropical regions where Annonaceae remains among the most poorly characterized families. Upon publication, the New Guinea checklist (Cámara-Leret et al. 2020) reported a total of 19 genera and 97 species of Annonaceae for the island. This is in stark contrast to other regions of Malesia which boast far greater diversity: Borneo (34 genera and 267 species; Turner 2011, POWO 2025), Peninsular Malaysia (36 genera and 238 species; POWO 2025), Sumatra (32 genera and 129 species; POWO 2025) and the Philippines (28 genera and 149 species; Pelser et al. continuously updated).

However, given the limited amount of taxonomic research historically conducted on the island, there is likely a high amount of diversity that remains undescribed or unrecorded. Only four genera in Papuasia have modern taxonomic treatments available, with the last three having been published within the last two years: *Pseuduvaria* Miq. (Su and Saunders 2006), *Xylopia* (Johnson and Murray 2023), *Friesodielsia* Steenis (Ezedin 2024), and *Monoon* Miq. (Ezedin 2025). In the last three works alone, a total of 22 new species were described, unveiling the need for further taxonomic work. Here, we contribute a revision to the genus *Pyramidanthe* Miq. for New Guinea.

Until recently, all the New Guinea species of the lianescent genus *Pyramidanthe* Miq. (Uvarieae: Fissistigmatinae) were treated under *Mitrella* Miq. Following a preliminary phylogenetic study based on six plastid loci, *Mitrella* was entirely subsumed under the formerly monotypic *Pyramidanthe* (Bangkomnate et al. 2021). Currently, the newly defined *Pyramidanthe* s.lat. encompasses 12 species distributed from southern Thailand to northern Australia (Bangkomnate et al. 2021, POWO 2025). The two genera were previously considered to be closely related due to several morphological similarities, including leaves with reticulated tertiary venation, outer petals with a basal excavation on the inner surface, and the inner petals being much smaller than the outer ones (Turner 2012; Bangkomnate et al. 2021). *Mitrella* had been primarily differentiated from *Pyramidanthe* by its flowers with 10–15 carpels (vs. 6), with 3–5 ovules each (vs. 6 or more), fruits with globose monocarps (vs. cylindrical), monocarps with smooth surface (vs. tuberculate), and sepals persistent in the fruit (Kessler 1993; Couvreur et al. 2012). However, the overall similarity between both genera, along with the challenges in finding reliable macromorphological differences between them, was difficult to reconcile (Turner 2012). In addition, both genera were found to form a single well-supported clade, sister to *Fissistigma* Griff. (Guo et al. 2017a, Bangkomnate et al. 2021); more recently, this clade was designated at subtribal level as Fissistigmatinae (Nge et al. 2024). Based on this preliminary set of morphological and molecular evidence, Bangkomnate et al. (2021) decided to combine *Mitrella* under *Pyramidanthe*. While we follow this decision here, additional phylogenomic studies are needed to provide further support for this union.

The synonymizing of *Mitrella* under *Pyramidanthe* caused a minor nomenclatural controversy. Since the two genera were described in the same work by Miquel (1865), both names had equal nomenclatural priority. The choice made by Bangkomnate et al. (2021) in favor of *Pyramidanthe*, established the priority of this name over *Mitrella* (ICN Art. 11.5, Turland et al. 2018). Following this, Turner (2022) proposed to conserve *Mitrella* over *Pyramidanthe*, arguing that the former name had been applied more widely across Malesia and Australia and thus required fewer combinations. The Nomenclatural Committee’s decision (Applеquist 2024: 292) was to reject Turner’s proposal in favor of retaining the name *Pyramidanthe* as the committee did not see the difference between the two as being “too great”, while also reluctantly noting that they believed Bangkomnate et al. (2021) “did not make the best choice”.

Currently, four species of *Pyramidanthe* are reported for New Guinea: *P. beccarii* (Scheff.) Bangk. & Chaowasku, *P. ledermannii* (Diels) Bangk. & Chaowasku, *P. schlechteri* (Diels) Bangk. & Chaowasku and *P. silvatica* (Diels) Bangk. & Chaowasku (Cámara-Leret et al. 2020, as *Mitrella*). Three of them were described during the last major treatment for the family in New Guinea, published more than a century ago (Diels 1912, 1915). In the present account, we analyzed all known specimens of the genus collected from New Guinea. Here, we describe four species as new and synonymize another. Descriptions, illustrations, and range maps are provided for all seven species of *Pyramidanthe* recognized, along with a key to those species.

The genus *Fissistigma*, sister to *Pyramidanthe* (Nge et al. 2024), is newly reported in New Guinea for the first time, with the species *F. latifolium* recorded from two collections originating from West Papua. Following the discovery of improper typification for the species, a neotype is designated. *Fissistigma* is currently known to range from Indochina to the Moluccas, with *F. latifolium* being the only species to reach as far east as the Moluccas (Turner 2018). With these new records, this genus’ range is extended further eastward into New Guinea.

Additionally, the genus *Desmos* Lour. (Uvarieae: Desminae) is reported in New Guinea for the first time. This follows the discovery of a single collection representing a new species, *D. insolens*. Overall, this account brings to 21 the total number of Annonaceae genera present in New Guinea, six of which are climbers. A key to the climbing genera of the region is provided.

## ﻿Materials and methods

We follow the concept of *Pyramidanthe* as proposed by Bangkomnate et al. (2021). We studied specimens of *Pyramidanthe*, filed under *Mitrella*, collected from the New Guinea region, as defined in Cámara-Leret et al. (2020). We examined a total of 91 specimen gatherings either digitally or in person from the following herbaria: A, AK, BO, BRI, BRIT, CANB, E, K, L, MIN, NSW, NY, SING, and US (acronyms following Thiers continuously updated). Species protologues were verified and digital images of the types for all known species were examined. The morphological terms largely follow Hickey (1973) for leaf shape and venation and Hewson (2019) for indument. Descriptions are based on dried material, unless otherwise noted. Due to morphological variation in the genus, our species delimitations here generally follow the “morphological cluster” species concept, often used in other morphologically complex genera (Knapp 2024). Notes on gatherings exhibiting unusual morphology are given where relevant. Conservation assessments, including calculations of Extent of Occurrence (EOO) and Area of Occupancy (AOO), were prepared using the GeoCAT tool (Bachman et al. 2011). The morphological keys presented here only apply to species present in the New Guinea region.

## ﻿Taxonomic treatment

### ﻿Key to the climbing genera of Annonaceae in New Guinea

**Table:** 

1	Tertiary veins indistinct from secondary veins, often raised on both surfaces; inflorescence peduncles hook-shaped; petals spoon-shaped	*** Artabotrys ***
–	Tertiary veins distinct from secondary veins, raised on one surface or not; inflorescence peduncles straight; petals not spoon-shaped	**2**
2	Indument of stellate hairs or mixed with simple; petals imbricate, subequal	*** Uvaria ***
–	Indument of simple hairs or glabrous; petals valvate, unequal (rarely subequal)	**3**
3	Inflorescences terminal and leaf-opposed, multi-flowered racemes or panicles	*** Fissistigma ***
–	Inflorescences axillary or supra-axillary (rarely leaf-opposed), single-flowered or fasciculate	**4**
4	Monocarps moniliform with distinct segmentation	*** Desmos ***
–	Monocarps not moniliform or sometimes irregularly constricted but without distinct segmentation	**5**
5	Twigs bearing lenticels; intersecondary veins absent; sepals not persistent in fruit; seeds always 1 per monocarp, globose	*** Friesodielsia ***
–	Twigs lacking lenticels; intersecondary veins usually present; sepals persistent in fruit; seeds 1–5 per monocarp, usually flattened on one or both sides	*** Pyramidanthe ***

### ﻿Key to the species of *Pyramidanthe* in New Guinea

**Table:** 

1	Twigs, petioles, and abaxial laminas covered in persistent rusty tomentum	**2**
–	Twigs, petioles, and abaxial laminas covered in sericeous hairs or glabrescent	**4**
2	Leaf apex acuminate; flowering pedicels 25–40 mm long, solitary; fruiting pedicels 20–35(–40) mm long	***P. montana***
–	Leaf apex usually acute or mucronate; flowering pedicels 1–8 mm long, solitary to fasciculate; fruiting pedicels 4–13(–40) mm long	**3**
3	Outer petals thin, chartaceous; intersecondaries prominently raised abaxially; abaxial surfaces sparsely tomentose and conspicuously glaucous	***P. nervosa***
–	Outer petals fleshy, (sub)coriaceous; intersecondaries usually inconspicuous; abaxial surfaces densely tomentose and inconspicuously glaucous	***P. beccarii***
4	Outer petals subglabrous, 14–20 cm wide, broadly triangular-ovate	***P. oblongifolia***
–	Outer petals densely sericeous, 4–12 cm wide, narrowly triangular-ovate	**5**
5	Leaves small, measuring up to 5 × 2.5 cm; outer petals 4–5 mm wide	***P. parvifolia***
–	Leaves large, measuring 6–22 × 3–7.5; outer petals 6–12 mm wide	**6**
6	Leaves generally broad, 4–6.5(–7.5) cm wide, apex usually cuspidate to mucronate or rarely acute to rounded; leaves and twigs often sparsely sericeous or early glabrescent; inflorescences usually 2–3-flowered; fruiting pedicels generally longer, 15–45 mm; occurring in lowland forests	***P. ledermannii***
–	Leaves generally narrow, 3–5 cm wide, apex variously acuminate to acute to obtuse to rounded; leaves and twigs often densely sericeous or late glabrescent; inflorescences single-flowered; fruiting pedicels generally shorter, 10–25 mm; occurring mostly in montane forests	***P. schlechteri***

### ﻿Revision of *Pyramidanthe* in New Guinea

#### ﻿1. Pyramidanthe beccarii

Taxon classificationPlantaeMagnolialesAnnonaceae

(Scheff.) Bangk. & Chaowasku, Willdenowia 51(3): 387. 2021.

0B43E739-B54C-5DEA-BADD-D657244D0425

Fig. 1C, D, G–J

Melodorum beccarii Scheff., Ann. Jard. Bot. Buitenzorg 2: 24. 1885 ≡ Mitrella beccarii (Scheff.) Diels, Bot. Jahrb. Syst. 49(1): 149. 1912 ≡ Fissistigma beccarii (Scheff.) Merr., Philipp. J. Sci. 15: 131. 1919. Type: [Indonesia], [West Papua], Andai, 1872, *Beccari 795* (lectotype, designated by Diels 1912, pg. 150: FI! [FI007574]; isolectotypes: A! [A00039457], B! [B 10 0325324], K! [K000574734]).
Melodorum beccarii var. lanceolatum Scheff., Ann. Jard. Bot. Buitenzorg 2: 25. 1881. Type: [Indonesia], [West Papua], Andai, 1872, *Beccari 593* (lectotype, designated by Diels 1912, pg. 149: FI-B! [FI007575] [Erb. Coll. Becc. 498]).

##### Type.

Based on *Melodorum beccarii* Scheff.

##### Description.

Woody climbers; twigs densely rusty tomentose. Leaves (thick) chartaceous; petioles 6–11 mm long, terete, deeply grooved, densely pubescent to glabrescent; laminas (3.5–)5–12.5(–23) × (1.8–)2–3.5(–5.5) cm, narrowly elliptic, lanceolate to narrow oblong, adaxial surface glabrous, dark green or olive-green glossy in vivo, abaxial surface densely rusty tomentose to pubescent, brownish gray or yellow (glaucous) in vivo and reddish brown in sicco, base acute, apex acute (to attenuate); venation eucamptodromous, primary vein sharply impressed above and raised below, secondary veins in (6–)8–12(–15) pairs, often inconspicuous above, spaced 6–10(–15) mm apart, angled at 55–70° to the midvein, intersecondaries frequent and prominent, tertiary veins irregularly percurrent to (sub)reticulate, inconspicuous to prominent above, very faint to prominent below. Inflorescences axillary, 1(–3)-flowered, pedicels 2–8 mm long; bracts 1–3, at the base of the pedicel, triangular ovate. Flowers creamy yellow to orange to brownish at anthesis; sepals 2–3 × 3–4 mm, partially connate at the base, broadly triangular ovate, slightly gibbous, outside densely rusty tomentose; outer petals 10–20 × 4–9 mm, narrowly triangular ovate, (sub)coriaceous, outside rusty tomentose, inside densely grey tomentose; inner petals 5 × 2 mm, broadly triangular ovate, connivent; stamens several, ca. 1 mm long; carpels ca. 10, 1.2–1.5 mm long, ovaries 0.8–1 cm long. Fruits consisting of 4–7 monocarps; pedicels 4–8(–12) mm long, sepals persistent; stipes 2–8 mm long; monocarps 9–12 × 5–12 mm, irregularly (sub)globose, rusty tomentose to glabrescent, yellowish to orangish when ripe. Seeds 2–3 per monocarp, light orangish brown, 8–10 × 2–4.5 mm, oblate, with rudimentary aril, testa smooth.

**Figure 1.**
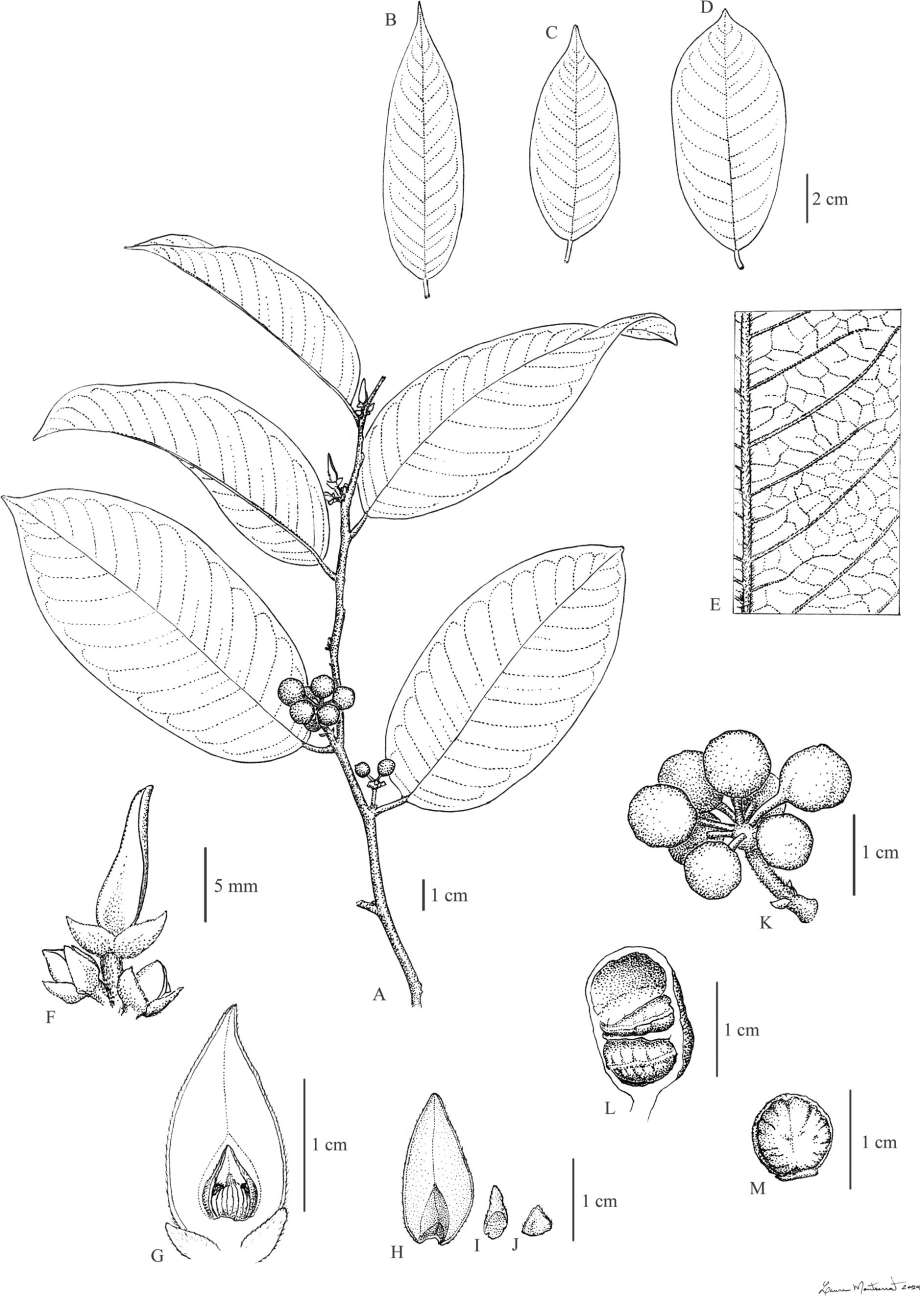
Rusty tomentose ‘beccarii’ group A, E–F, K. *Pyramidanthe nervosa* Ezedin & J.C.Lopes. A. Branch with fruits; E. Detail of the leaf venation; F. Flowers and flower buds; K. Fruit. B, L–M. *Pyramidanthe montana* Ezedin & J.C.Lopes. B. Leaf; L. Monocarp opened, showing inner cavity with one seed removed; M. Seed. C, D, G–J. *Pyramidanthe beccarii* (Scheff.) Bangk. & Chaowasku. C, D. Variation in leaf size and morphology; G. Flower with one outer petal removed; H. Outer petal; I. Inner petal; J. Sepal. A, E, K. from *de Vogel 9651*. B, L–M. From *Kalkman 5321*. C, G–J. from *Kerenga LAE 56551*. D. from *Takeuchi & Kulang 11449*. F. from *Moll 9772*. Illustrated by L. Montserrat.

##### Distribution

**Figure 2.**
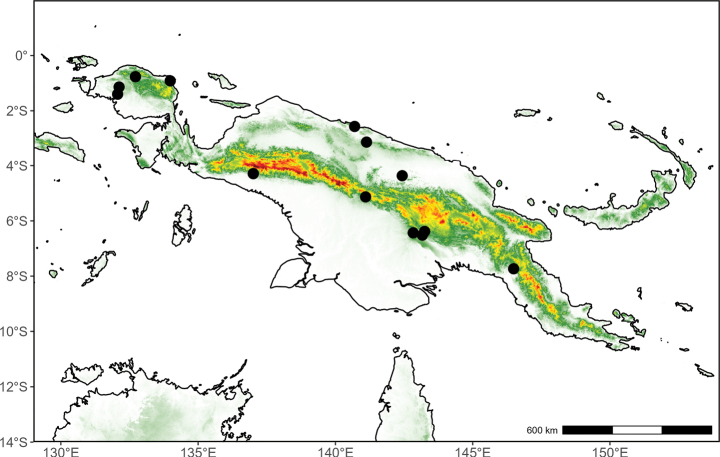
Distribution map of *Pyramidanthe beccarii*.

**(Fig. 2).** Indonesian New Guinea and Papua New Guinea.

##### Ecology and habitat.

In primary lowland to lower montane forests, up to ca. 1000 m. Commonly collected in riverine and gallery forests; also found on limestone.

##### Vernacular names.

Magisum (Kutubu), Bibka (Manikiong). Akovah (Maibrat).

##### Preliminary IUCN conservation status.

Least Concern (LC). The species’ widespread distribution effectively shields it from threats. Its EOO is more than 340,000 km^2^ and its AOO is 52 km^2^ with a standard cell width of 2 km.

##### Notes.

Within the concept of *P. beccarii* adopted here, a few collections appear to deviate slightly from the type. The indument of *Schodde 2442* and *Wiakabu et al. LAE 50607* presents as golden-orangish colored and not as densely covering the abaxial laminas. The specimen *Takeuchi & Kulang 11449* approaches *P. nervosa* with its larger leaves and more abaxially prominent secondaries; however, all other features align it with *P. beccarii*, namely the thicker outer petals that are not as tightly constricted. *Utteridge 330* differs primarily in its sparser tomentum on the abaxial laminas which are also glaucous and with conspicuous venation.

The specimen *Gjellerup 460* bears the longest leaves at up to 23 × 5.5 cm whereas all other specimens max out at around 12 cm in length. This length is similar to that of the synonymized variety M. beccarii var. lanceolata, whose type bears leaves up to 13 cm long. While this length may appear unusual, both the varietal type (*Beccari 593*) and *Gjellerup 460* also display leaves as small as 5.5 × 3 cm, falling within the range of *P. beccarii*. It could be that there is variability in leaf length, as is often seen in lianas, perhaps brought about by local environmental factors. Flowers of both remain consistent with those of *P. beccarii*.

Turner (2018) did not list the variety as having been lectotypified by Diels (1912).

##### Additional specimens examined.

Indonesia. **Papua** • Humboldt Bay [=Yos Sudarso Bay, Jayapura], [ca. 2°34'S, 140°42'E], [ca. sea level], 1911, *Gjellerup 460* (BO [BO-1350060], L [L.1754300]) • PT Freeport Indonesia project area, in terrace above Otomona River and on slopes above terrace, towards Mile 50, 4°17'30"S, 137°0'30"E, 200–300 m, 12 Apr 2000, *Utteridge 330* (A [A02607265], BO [BO-1425885], K [K000260883], L [L.3728216], SING); **West Papua** • Vogelkop Peninsula, Ewai (E of Teminaboean [= Teminabuan]), [ca. 1°24'S, 132°04'E], 200–250 m, 13 May 1958, *Versteegh BW 7440* (BO [BO-1372538]) • Vogelkop Peninsula, Ije River valley, central part of the Tamrau Ra. [=Tambrauw Mountains], S slope, path from Sudjak village [=Sedjak], to Mt. Kusemun, Aiwa R., [ca. 0°46'S, 132°43'E], 840 m, 7 Nov 1961, *van Royen & Sleumer 7717* (A [A02263500], BO [BO-1372537]) • Surroundings of Ayawasi, [ca. 1°08'24"S, 132°07'12"E], [ca. 100 m], 9 Nov 1995, *Ridsdale 2202* (L [L.1757507, L.1757508]).

Papua New Guinea. **East Sepik** • Sepikgebiet [=Sepik region], [bivouac 18, on a tributary of the April River], [ca. 4°22'S, 142°26'E], 165 m, Nov–Dec 1912, *Ledermann 9790* (L [L.1754301]); **Gulf** • Lakekamu, east branch of the Avi Avi River, secondary streambed above the track between basecamp and Tekedu, 7°44'S, 146°29.5'E, 105 m, 26 Oct 1996, *Takeuchi & Kulang 11449* (A [A02263505], BRI [AQ0643115], BRIT [BRIT619080, BRIT619079], E [E00181764], MIN [974143], NSW [NSW473911], NY [NY02729231, NY4726774, NY4726775], US [US3385639]); **Sandaun** • Mt. Yungat, Nth slopes Bewani Mts., Bewani subprov., 5°08'S, 141°06'E, 1030 m, 20 Sep 1982, *Kerenga LAE 56551* (A [A02263498], L [L.1757537], NSW [NSW453629]) • Meinat flood plain N slopes Bewani, Mts. 11 km SSW of Bewani, 3°08'S, 141°08'E, 300 m, 22 Sep 1982, *Wiakabu et al. LAE 50607* (A [A02263499], BRI [AQ0369160], LAE [264880], NSW [NSW466844]); **Southern Highlands** • Along Soro River, Lake Kutubu, [ca. 6°23'S, 143°15'E], 760 m, 7 Oct 1961, *Schodde 2442* (A [A02263502], LAE [54188, 2 sheets]) • Mt. Bosavi, northern side, near the mission station, 700–800 m, ± 6°26'S, 142°50'E, 5 Oct 1973, *Jacobs 9020* (L [L.1757498]) • Waro airstrip, 20 km SSW of Kutubu, 6°31'S, 143°10'E, 500–600 m, 13 Oct 1973, *Jacobs 9199* (BO [BO-1372534], L [L.1757532], US [US03900187]).

#### ﻿2. Pyramidanthe ledermannii

Taxon classificationPlantaeMagnolialesAnnonaceae

(Diels) Bangk. & Chaowasku, Willdenowia 51(3): 389. 2021.

B87BF646-62AF-594D-A94F-2FB9DA520828

Fig. 3A–C

Mitrella ledermannii Diels, Bot. Jahrb. Syst. 52: 183. 1915. Type: [Papua New Guinea], Sepik region [=East Sepik], [bivouac above Malu], 19 Mar 1912, *Ledermann 6672* (lectotype, designated by Kessler et al. 1995, pg. 39: B! [10 0325315]; isolectotype: K! [K000574731, K000574733]).

##### Type.

Based on *Mitrella ledermannii* Diels.

##### Description.

Woody climbers; twigs sparsely sericeous to glabrous. Leaves coriaceous; petioles (8–)10–16(–22) mm long, terete, deeply grooved, (densely) sericeous to glabrescent; laminas (7–)10–17(–22) × 4–6.5(–7.5) cm, narrowly elliptic to elliptic, rarely narrow obovate, adaxial surface sparsely sericeous to glabrescent, dull dark green in vivo, dark greyish-brown in sicco, abaxial surface (densely) sericeous, dull green, reddish-brown in sicco, base obtuse to rounded, rarely acute, apex (acute to) cuspidate to mucronate (to rounded); venation eucamptodromous, primary vein impressed above and raised below, secondary veins in (6–)9–16 pairs, spaced 9–17 mm apart, angled at 60–75° to the midvein, intersecondaries frequent, tertiary veins (sub)reticulate, usually inconspicuous, sometimes bifacially raised. Inflorescences axillary, (1–)2–3-flowered, pedicels 10–23 mm long; bracts 2–3, near the base of the pedicel, (narrowly) triangular ovate, often persistent. Flowers yellowish to velvety brown at anthesis; sepals 3–3.5 × 3 mm, connate at the base, broadly triangular ovate, slightly gibbous, outside (densely) sericeous; outer petals (12–)15–21 × 8–12 mm, narrowly triangular ovate, thick coriaceous, outside sericeous, inside densely grey tomentose; inner petals 3–4 × 3 mm, triangular ovate, outside mostly glabrous except the puberulous apex, inside glabrous; stamens several, ca. 1 mm long; carpels ca. 6, ca. 1 mm long. Fruits consisting of 3–13 monocarps; pedicels 15–30(–45) mm long, sepals persistent; stipes (6–)8–15(–20) mm long; monocarps (5–)9–14(–20) × (6–)8–10(–13) mm, globose to oblongoid, the apex rarely rostrate, glabrous, brownish pink to orange brown when ripe. Seeds ca. 3 per monocarp, glossy dark brown, 7.5–9(–14) × 3.5–5(–9) mm, oblate or hemi-ellipsoidal, with fleshy rudimentary aril, testa smooth.

**Figure 3.**
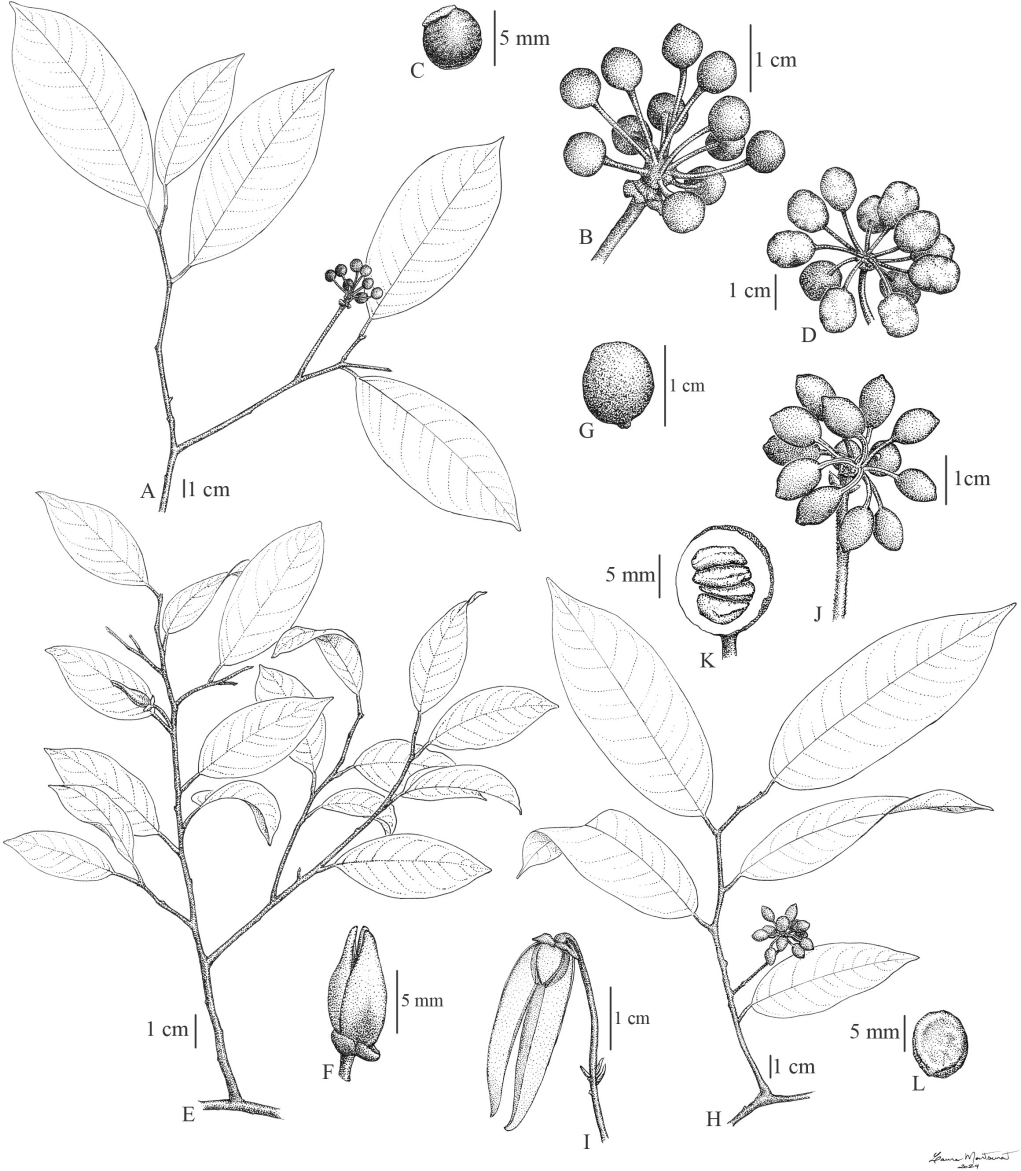
Sericeous-subglabrous ‘schlechteri’ group. A–C. *Pyramidanthe ledermannii* (Diels) Bangk. & Chaowasku. A. Branch with fruit; B. Fruit; C. Seed. D–G. *Pyramidanthe parvifolia* Ezedin & J.C.Lopes. D. Fruit; E. Branch with flower; F. Flower; G. Monocarp. H–L. *Pyramidanthe schlechteri* (Diels) Bangk. & Chaowasku. H. Branch with fruit; I. Flower with one outer petal removed; J. Fruit; K. Monocarp opened, showing seed arrangement; L. Seed. A–C from *Henty & Foreman NGF 42681*, D–G from *Vinas & Wiakabu LAE 59640*, H, J from *Henty & Foreman NGF 42681*, I from *Hartley 11488*, K–L from *Clemens 3850*. Illustrated by L. Montserrat.

##### Distribution

**Figure 4.**
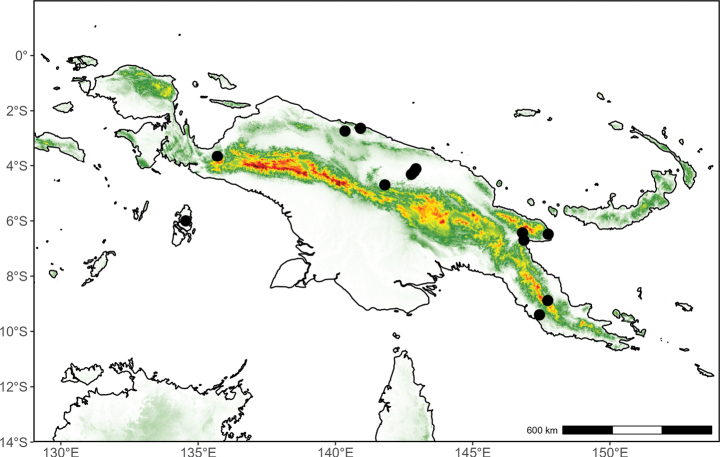
Distribution map of *Pyramidanthe ledermannii*.

**(Fig. 4).** Indonesian New Guinea and Papua New Guinea.

##### Ecology and habitat.

In lowland forests, mostly up to ca. 800 m. One of the specimens (*Clemens 41552*) reports the elevation as “2500–4500 ft” (762–1371 m). It is more likely that this specimen was collected on the lower end of that spectrum, likely around 2500 ft, especially since another specimen from a nearby location (*Clemens 1199*) was collected at 2500 ft. Thus, the higher end of this elevation is not considered in the elevational range for this species, the maximum being estimated at 800 m. The flowers are apparently fragrant (*Carr 16220*). The label of *Clemens 41552* notes the specimen as being the host of the rust fungus, *Sphaerophragmium boanense* Cummins.

##### Preliminary IUCN conservation status.

Least Concern (LC). Its broad distribution across the lowlands effectively shields it from any potential threat. The EOO is over 600,000 km^2^ and the AOO 52 km^2^ with a standard cell width of 2 km.

##### Notes.

This species is close to *P. schlechteri* due to its sericeous indument and could potentially form part of a larger *P. schlechteri* complex. However, *P. ledermannii* is maintained here on account of its morphology and elevational range, which form a recognizable unit. The morphological differences which allow for the recognition of *P. ledermannii* as a separate taxon from *P. schlechteri* include the following: generally larger and coriaceous laminas with acute to rounded apices (vs. generally elliptic and chartaceous with acuminate apices), leaves and twigs more often early glabrescent or glabrous (vs. often densely sericeous or late glabrescent), inflorescences usually 2–3 flowered (vs. single-flowered), fruiting pedicels generally longer at 15–45 mm (vs. 10–25 mm), and being restricted to lowland forests (vs. mostly montane).

##### Additional specimens examined.

Indonesia. **Maluku** • Aroe Islands [=Aru Islands], P. Kobroor, Dosinamaloe [= Dosinamalau], [ca. 6°00'S, 134°33'E], [sea level], 30 May 1938, *Buwalda 5101* (BO [BO-1385470], L [L.1757565]); **Papua** • Patema, 40 km inward of Nabire, [ca. 3°39'S, 135°42'E], 300 m, 6 Mar 1940, *Kanehira & Hatusima 12394* (BO [BO-1362067]) • Res. Hollandia [=Jayapura], mouth of the river Tami, [ca. 2°38'S, 140°55'E], [sea level], 20 Mar 1956, *Kalkman BW 3421* (A [A02263529], BO [BO-1408162], L [L.1757551], LAE [14773, 2 sheets]) • Sekoli, South of Lake Sentani, [ca. 2°44'S, 140°21'E], 75 m, 18 Feb 1960, *Schram BW 9393* (L [L.1757575], LAE [213326, 2 sheets]).

Papua New Guinea. **Central** • Koitaki, [ca. 9°24'S, 147°26'E], 457 m, 4 May 1935, *Carr 12153* (A [A02263538], SING); **East Sepik** • Kaiserin Augusta-Fluss [=Sepik River], [ca. 4°13'S, 142°52'E], [ca. near sea level], Dec–Feb 1913, *Ledermann 10781* (K [K000574732]) • Along Yapa (Hunstein River), Ambunti subdistrict, [ca. 4°19'S, 142°46'E], 122 m, 21 Jul 1966, *Hoogland & Craven 10628* (A [A02263532], BRI [AQ0351315], L [L.1757548, L.1757549], LAE [149522, 2 sheets]); **Morobe** • Quembung Mission, [ca. 6°29'S, 147°45'E], 762 m, 10 Dec 1935, *Clemens 1199* (A [A02263539], L [L.1757480]) • Boana, [ca. 6°26'S, 146°49'E], 762–1371 m, May–Nov 1940, *Clemens 41552* (A [A02263534]) • Markham-Oomsis Ridge, 13 km of Lae, 6°42'S, 146°52'E, 100 m, 13 Oct 1982, *Streimann 8617* (A [A02263524], CANB [CBG8314364.1], L [L.1757536], LAE [250100]); **Oro** • Kokoda, [ca. 8°53'S, 147°44'E], 366 m, 24 Mar 1936, *Carr 16220* (NY [NY04726780], SING) • ibid., 24 Mar 1936, *Carr 16221* (L [L.1757539], NY [NY04726779], SING); **Sandaun** • Kokomo Creek, tributary of Freida RIver, Telefomin Distr., 4°42'S, 141°48'E, 490 m, 29 Jun 1969, *Henty & Foreman NGF 42681* (A [A02263515], BO [BO-1455962], BRI [AQ0211277], L [L.1757511], SING).

#### ﻿3. Pyramidanthe montana

Taxon classificationPlantaeMagnolialesAnnonaceae

Ezedin & J.C.Lopes sp. nov.

483E80CC-622C-5268-AF94-25AAC02EE8A3

urn:lsid:ipni.org:names:77369117-1

Fig. 1B, L, M

##### Diagnosis.

Differs from *P. beccarii* in its thicker coriaceous laminas (vs. chartaceous), longer flowering pedicels measuring 25–40 mm long (vs. 2–8 mm), longer fruiting pedicels measuring 20–40 mm long (vs. 4–12 mm), and sepals that are usually lost at maturity (vs. persistent in fruit).

##### Type.

Papua New Guinea. Southern Highlands • Anthropogenic grassland with scattered regrowth patches foothills above expedition’s Beneria River basecamp, 6°03'11"S, 142°58'55"E, 1425 m, 8 May 2005, *Takeuchi et al. 19557* (holotype: A! [A02263525]; isotypes: K! [K003818149], L! [L.2067322, L.2067323], LAE! [285550, 2 sheets]).

##### Description.

Woody climbers; twigs rusty tomentose, glabrescent. Leaves (sub)coriaceous; petioles (5–)8–10(–12) mm long, terete, grooved, tomentose; laminas (6–)8–14.5 × (2.5–)3–5 cm, oblong to narrowly elliptic, adaxial surface (densely) white-grey sericeous and very early glabrescent often with few sparse hairs along midrib remaining, dark shiny green in vivo and greyish brown in sicco, abaxial surface (sparsely to) densely rusty tomentose, brownish-green, base obtuse to rounded, rarely acute, apex acuminate; venation eucamptodromous (to brochidodromous), primary vein impressed above and raised below, secondary veins in 5–10 pairs, spaced 9–18 mm apart, angled at 55–70° to the midvein, intersecondaries frequent, tertiary veins (sub)reticulate (to irregularly percurrent), bifacially inconspicuous. Inflorescences axillary, 1-flowered, flowering pedicels 25–40 mm long; bracts 2–3, near the base of the pedicel, triangular ovate, sometimes persistent or leaving a scar when caducous. Flowers golden at anthesis, sepals 3–4 × 2 mm, connate at the base, broadly triangular ovate, slightly gibbous, outside rusty tomentose to glabrescent, inside densely white puberulous; outer petals 10–16 × 5–7 mm, (broadly) triangular ovate, coriaceous, outside densely rusty tomentose, inside densely grey tomentose; inner petals 6 × 3–4 mm, triangular ovate, outside mostly glabrous except the puberulous apex, inside glabrous except for the pubescent apex; stamens several, ca. 1.5 mm long; carpels ca. 10, ca. 2 mm long. Fruits consisting of 4–13 monocarps; pedicels 20–35(–40) mm long, densely tomentose (to glabrescent), sepals usually lost by maturity; stipes (2–)6–8 mm long, sometimes appearing subsessile; monocarps 9–15(–25) × 8–11 mm, globose to oblongoid, the apex often slightly rostrate, glabrous, yellow to light orange when ripe. Seeds 1–4 per monocarp, glossy light to dark brown, 8–9 × 3–5 mm, oblate or hemi-ellipsoidal, with fleshy rudimentary aril, testa smooth.

##### Etymology.

After its montane distribution.

##### Distribution

**Figure 5.**
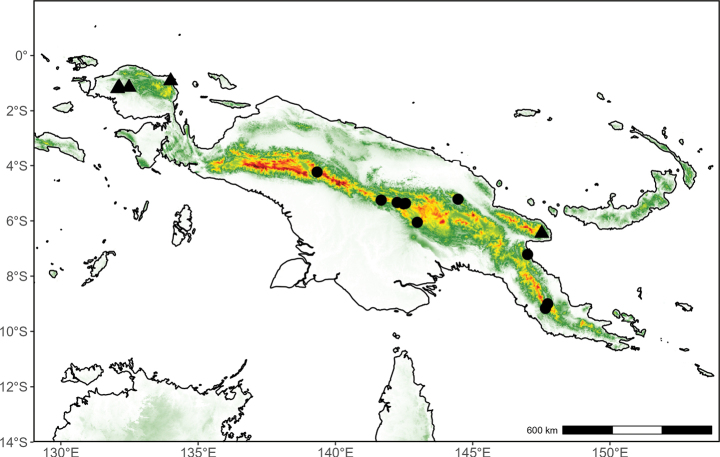
Distribution map of *Pyramidanthe montana* (circle) and *P. nervosa* (triangle).

**(Fig. 5).** Indonesian New Guinea and Papua New Guinea.

##### Ecology and habitat.

In lower to mid montane forests, including both primary and secondary forests along with other disturbed sites, from 1280–1900 m.

##### Vernacular names.

Binelna (Oksapmin), Jop (Kalam).

##### Preliminary IUCN conservation status.

Least Concern (LC). Its EOO is over 130,000 km^2^ and AOO 40 km^2^ with a standard cell width of 2 km. It is likely to be found elsewhere in the Central Cordillera and other montane regions on the island.

##### Notes.

Similar in appearance to *P. beccarii*, this species is defined by its elliptic leaves with a distinctly tapering acuminate apex, fewer secondaries that are spaced more widely apart, and most notably its longer flowering and fruiting pedicels.

##### Additional specimens examined.

Indonesia. **Papua** • Snow Mountains Region, E of the Baliem River Valley, Kab. Jayawijaya, Kec. Kurima, 4°14'S, 139°20'E, 1650 m, Oct 1992, *Milliken 1404* (BO [BO-0014979], K [K003818174]).

Papua New Guinea. **Central** • Isuarava, [ca. 9°00'S, 147°44'E], ca. 1520 m, 12 Feb 1936, *Carr 15543* (A [A02263503, A02263504], L [L.1757477], NY [NY4726394], SING [3 sheets]) • Port Moresby subdistrict, West of Efogi, 1520 m, 9°10'S, 147°39'E, 26 Sep 1973, *Foreman et al. LAE 60013* (BRI [AQ0350323], L [L.1757544], LAE [210641]); **Hela** • 4 miles from Kopiago on Koroba Rd., Lake Kopiago Sub-Dist., 5°22'S, 142°33'E [=ca. 5°23'S, 142°29'E], 1508 m, 2 Nov 1968, *Vandenberg et al. NGF 39974* (L [L.1757500]) • 2 miles from Kopiago on Paga Hill Rd., near Lutheran Misison, Lake Kopiago Sub-Dist., 5°22'S, 142°33'E, 1280 m, 5 Nov 1968, *Vandenberg et al. NGF 42013* (A [A02263517], L [L.1757514], LAE [106089]); **Madang** • Schrader Range, Kaironk Valley, 5°13'S, 144°28'E, ca. 1600 m, 1 Nov 1993, *Gardner 7200* (AK [AK340549], L [L.1757501]); **Morobe** • Near Skindewai, Wau–Mubo road, [ca. 7°13'S, 147°00'E], 1645 m, 5 Jan 1956, *Womersley & Millar NGF 8342* (A [A02263501], BRI [AQ0211472], L [L.1757506], LAE [9013]); **Sandaun** • Between Feramin and Telefomin, Hindenburg Range, Telefomin subdist., 5°15'S, 141°40'E, 1500 m, 18 Sep 1966, *Kalkman 5321* (A [A02263497], K [K003818147], LAE [95181]) • Oksapmin, Telefomin subdist., 5°20'S, 142°15'E, 1890 m, 16 Oct 1968, *Henty et al. NGF 41579* (L [L.1757515], LAE [107313]).

#### ﻿4. Pyramidanthe nervosa

Taxon classificationPlantaeMagnolialesAnnonaceae

Ezedin & J.C.Lopes sp. nov.

BEBA4113-A98B-5C69-B603-BC036F0082F4

urn:lsid:ipni.org:names:77369120-1

Fig. 1A, E, F, K

##### Diagnosis.

Differs from *P. beccarii* in its broadly elliptic leaves (vs. narrowly elliptic), usually mucronate apex (vs. acute to attenuate), secondary veins usually in 12–15 pairs (vs. 6–12 pairs), abaxial laminas that are conspicuously glaucous with prominent secondaries and intersecondaries, and generally thinner outer petals.

##### Type.

Indonesia. West Papua • Oemboei, near Andai, SW of Manokwari, [0°55'S, 133°58'E], ca. 50 m, 21 May 1960, *Moll BW 9772* (holotype: L! [L.1757513]; isotypes: BO! [BO-1349452], LAE! [73853, 2 sheets], WAG! [WAG.1494740, WAG.1494741]).

##### Description.

Woody climbers; twigs densely rusty tomentose and often intermixed with longer dark-colored pubescent hairs. Leaves (sub)coriaceous; petioles 10–16 mm long, terete, deeply grooved, densely tomentose-pubescent; laminas (9–)11–14(–18) × (3.5–)5–7(–9) cm, elliptic (to ovate or obovate), adaxial surface white sericeous to early glabrescent, greyish brown in sicco, abaxial surface densely rusty tomentose-pubescent, glaucous (blue-green) white in sicco, base (acute to) rounded to obtuse (to truncate), apex mucronate (to acute); venation eucamptodromous (to brochidodromous), primary vein impressed above and raised below, secondary veins in (9–)12–15 pairs, spaced (6–)8–11 mm apart, angled at 40–45° to the midvein, often with two orders of prominent intersecondaries, 1° intersecondaries always present and prominent, 2° intersecondaries often present but less prominent and located between the secondaries and the 1° intersecondaries, tertiary veins irregularly (straight) percurrent to subreticulate, abaxially prominent. Inflorescences axillary, (2–)3–5-flowered, pedicels 1–3 mm long; bracts 1, near the base of the pedicel, narrowly triangular-ovate, often persistent. Flowers yellowish cream to brownish green at anthesis; sepals 4 × 2 mm, connate at the base, broadly triangular ovate, slightly gibbous, outside rusty sericeous; outer petals 10–15 × 2.5–4.5 mm, narrowly triangular ovate, chartaceous, outside densely rusty sericeous, inside densely grey tomentose; inner petals 5 × 2 mm, triangular ovate, outside sparsely pubescent and becoming more densely so towards the apex, inside glabrous; stamens several, ca. 1 mm long; carpels ca. 10, ca. 2 mm long, ovaries ca. 1 mm long, with tufts of appressed hair at the base. Fruits consisting of 2–10 monocarps; pedicels 5–13(–40) mm long, sepals persistent; stipes 1–5 mm long; monocarps 8–11 × 6–9 mm, (irregularly) globose to ovoid, often irregularly raised or bumpy, glabrous, yellowish to brown when ripe. Seeds 1–3 per monocarp, brown, 6 × 4 mm, oblate, with fleshy rudimentary aril, testa somewhat rough.

##### Etymology.

After the prominent venation of the abaxial laminas.

##### Distribution

**(Fig. 5).** Indonesian New Guinea and Papua New Guinea.

##### Ecology and habitat.

Found in lowland forests up to ca. 600 m.

##### Vernacular names.

Aa-safe, Aa-amos (Karel Yumte [=Maybrat?]), Bibka (Manikiong).

##### Preliminary IUCN conservation status.

Least Concern (LC). The rather distinct collection of *Hoogland 8867* from the Huon Peninsula extends the EOO to more than 90,000 km^2^ with an AOO of 20 km^2^. If this specimen were to be excluded, however, the species would effectively be restricted to the Vogelkop Peninsula with an EOO of 756 km^2^ and an AOO of 16 km^2^ thus giving it a status of Endangered (EN).

##### Notes.

This group forms a recognizable morphological unit that is closely related to *P. beccarii* due to the dense rusty tomentum. At times, there may appear to be some morphological overlap between *P. beccarii* and *P. nervosa*, primarily in instances where leaves of the former have more conspicuous venation. Nonetheless, the outer petals of *P. beccarii* are thicker, and the flowers are usually solitary or in fascicles of up to three (vs. usually three or more).

It should be noted that *Hoogland 8867* deviates from the rest in its fruiting pedicels which are much longer at 40 mm. Nonetheless, it is tentatively placed here for now as the laminar features agree with the concept of *P. nervosa*.

##### Additional specimens examined.

Indonesia. **West Papua** • Ayawasi, Kec. Aifat, Kab. Sorong, first hill NE of the behind the asrama, 1°09'S, 132°29'E, 450 m, 7 Sep 1995, *de Vogel 9651* (L [L.4462592]) • SW of Ayawasi, Irafe, plot 12, [ca. 1°12'S, 132°04'E], 480 m, 26 Feb 1996, *Polak 1075* (L [L.1754955, L.1754956]) • SW of Ayawasi, near Sakof rukam, near plot 11, 1°08'24"S, 132°07'12"E, 475 m, 4 May 1996, *Polak 1245* (K [K001870525], L [L.1757473, L.1757474]).

Papua New Guinea. **Morobe** • Masba Creek Area, c. 3 miles S of Pindiu, Huon Peninsula, [ca. 6°27'S, 147°31'E], 610 m, 5 May 1964, *Hoogland 8867* (A [A02263507, A02263506], L [L.1757517, L.1757518], LAE [108661], US [US03900002, US03900004]).

#### ﻿5. Pyramidanthe oblongifolia

Taxon classificationPlantaeMagnolialesAnnonaceae

Ezedin & J.C.Lopes sp. nov.

BB64B48E-8985-5BE2-9AD8-10F1A42C0A21

urn:lsid:ipni.org:names:77369121-1

Fig. 6

##### Diagnosis.

Differs from *P. schlechteri* in bearing triangular-ovate outer petals at 14–20 mm wide (vs. 6–10 mm) with sparsely sericeous to glabrescent outside (vs. densely rusty sericeous), and shorter stipes 4–6 mm long (vs. 6–12 mm).

##### Type.

Papua New Guinea. Morobe • Kamiali Wildlife Management Area, banks of Saia River near Hessen Bay, sea level, near 7°21.7'S, 147°08.3'E, 21 Jul 2000, *Takeuchi 14693* (holotype: A! [A02263513]; isotypes: K! [K003818169], L! [L.1757470], LAE! [277040, 2 sheets], US! [US3449680]).

##### Description.

Woody climbers; twigs sparsely sericeous, very early glabrescent. Leaves coriaceous; petioles (5-)6–10 mm long, terete, deeply grooved, glabrous; laminas 5–11.5(–17) × (1.5–)2–4 cm, (elliptic to) narrowly oblong (to lorate), adaxial surface sparsely sericeous and very early glabrescent, dark green to yellow green in vivo and grayish to reddish brown in sicco, abaxial surface sparsely sericeous and very early glabrescent, glaucescent light green to blue-green in vivo and greyish to reddish brown in sicco, base obtuse to rounded or truncate (to subcordate), apex acute to obtuse (to slightly acuminate); venation eucamptodromous (to brochidodromous), primary vein (shallowly) impressed above and raised below, secondary veins in 7–12 pairs, spaced 7–15 mm apart, angled at (55–)60–75° to the midvein, rather faint, intersecondaries frequent and faint, tertiary veins irregularly (sub)reticulate, often bifacially prominent. Inflorescences axillary, 1-flowered, pedicels 20–30 mm long; bracts 2, near the base of the pedicel, triangular-ovate, early caducous. Flowers yellow to orange-yellowish at anthesis; sepals 4 × 3.3 mm, connate at the base, broadly triangular ovate, slightly gibbous, outside glabrous to slightly puberulous, inside densely white puberulous; outer petals 20–37 × 14–20 mm, broadly triangular-ovate, subcoriaceous, outside densely verruculose and sparsely sericeous to glabrescent, inside sparsely yellow-orange tomentose; inner petals 7–11 × 6 mm, triangular-ovate, outside glabrous except for the densely puberulous apex, inside glabrous except for the apex; stamens several, 2–3 mm; carpels 5–17, ca. 2 mm long, ovaries ca. 1 mm long. Fruits consisting of 3–6 monocarps; pedicels (10–)35–40 mm long, sepals persistent; stipes 4–6 mm long; monocarps 11–15(–24) × 7–10 mm, ovoid to oblongoid, often irregularly raised or bumpy, often with a rostrate to rounded apex, glabrous, yellow to orange when ripe. Seeds 1–4 per monocarp, glossy dark (purplish) brown, 6–8 × 3 mm, oblate or hemi-ellipsoidal, with fleshy rudimentary aril, testa smooth.

**Figure 6.**
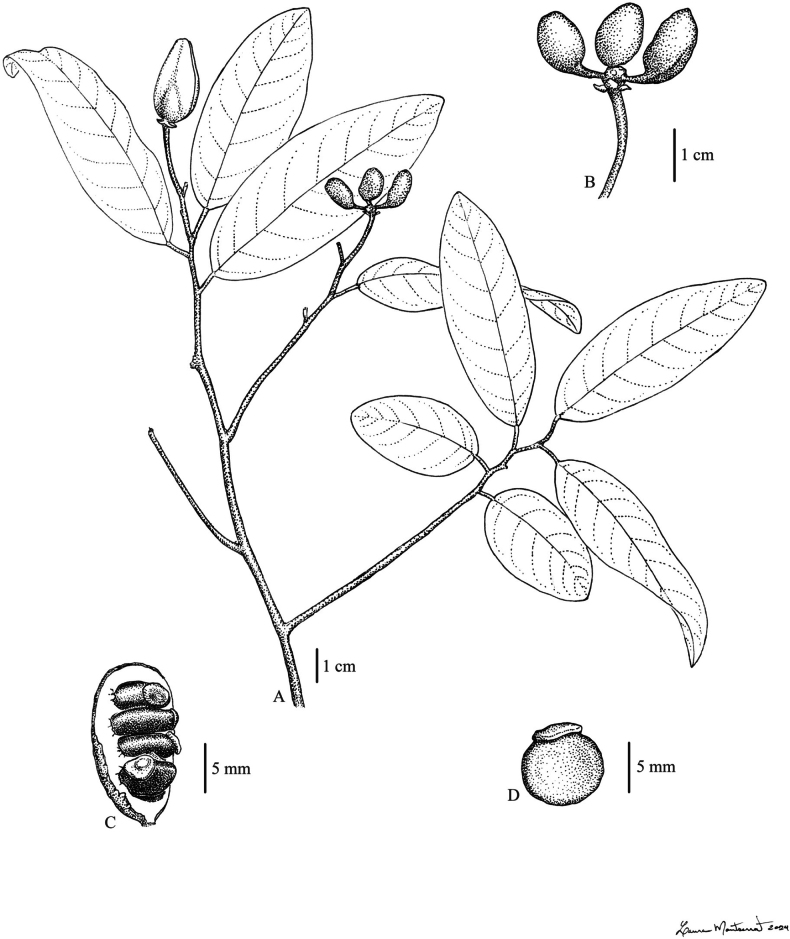
*Pyramidanthe oblongifolia* Ezedin & J.C.Lopes. A. Branch with flower and fruit; B. Fruit; C. Monocarp opened, showing disposition of the seeds; D. Seed. A–B from *Takeuchi 14693*, C–D from *Takeuchi 9235*. Illustrated by L. Montserrat.

##### Etymology.

After its distinctly oblong laminas.

##### Distribution

**Figure 7.**
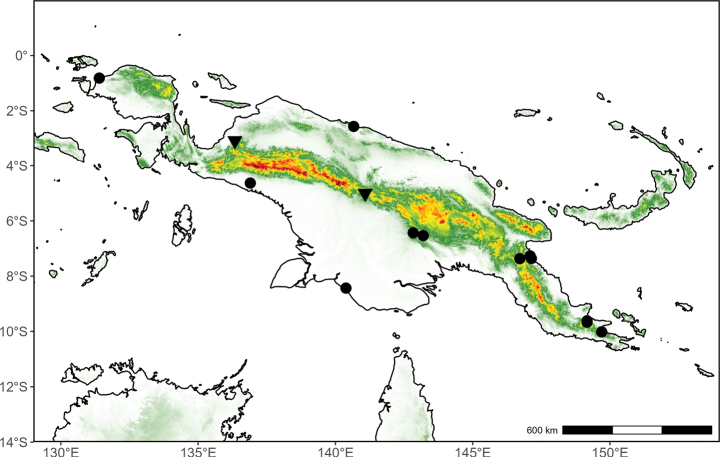
Distribution map of *Pyramidanthe oblongifolia* (circle) and *P. parvifolia* (triangle).

**(Fig. 7).** Indonesian New Guinea and Papua New Guinea.

##### Ecology and habitat.

From lowland to lower montane forests, up to ca. 900 m.

##### Vernacular names.

Kiesenaigiek (Mooi).

##### Preliminary IUCN conservation status.

Least Concern (LC). This species is widespread across the island with an EOO of over 700,000 km^2^ and AOO of 48 km^2^.

##### Notes.

This species can be readily identified by its distinctly oblong leaves, large outer petals, long flowering pedicels, and monocarps that are often cylindrical with a rostrate apex. The narrowly oblong leaf shape, as the specific epithet indicates, is unique to this species, with only a couple of collections of *P. schlechteri* bearing similarly shaped laminas. Due to limited flowering material, the flowers could not be dissected to observe the inner chamber. Instead, the parts of the inner flower chamber were described from the detailed field notes of *Takeuchi 9235* and *14693*; thus, their measurements are representative of fresh material.

This species was referred to as two separate species, *Mitrella* sp. 1 (*Takeuchi et al. 14192*) and *Mitrella* sp. 2 (*Coode 8031*), in the phylogenetic analyses by Bangkomnate et al. (2021). Both samples appeared as strongly supported sisters in the tree, this clade then sister to *Utteridge 330*, here placed under *P. beccarii*.

The collection of *Jacobs 8788* (L!) from the middle slopes of Mt. Bosavi superficially resembles this species, which is also known to occur in the area at lower elevations. However, it differs notably as it has densely rusty tomentose twigs and lamina undersides. Due to the uncertain placement of this specimen, along with its lack of flowers, it is excluded from this treatment. It may represent a separate taxon, but the material is too insufficient to warrant description at this time. Also excluded from this account is *Clemens & Clemens 498* (L!), which is superficially similar to this species but it is sterile and with notably larger leaves.

##### Additional specimens examined.

Indonesia. **Papua** • Soengei Maroka [=?Sungai Merauke (=Maro River)], [ca. 8°26'S, 140°23'E], [sea level], Apr 1901, *Jaheri 340* (BO [BO-1353934, BO-1353935, BO-1353936]) • Upper part of Hollandia (=Sukarnapura =Djajapura [=Jayapura]), [ca. 2°34'S, 140°40'E], 300 m, 26 Jul 1966, *Kostermans & Soegeng 37* (BO [BO-1385472]) • Mimika Regency, PT-Freeport Indonesia Concession Area, along main road from Portsite to Timika, km 23, 4°38'S, 136°54'E, 10 m, 16 Feb 1998, *Coode 8031* (A [A02263511, A02263512], BISH [BISH1298086], L [L.3724685, L.3728214, L.3724684], LAE [284763], SING); **West Papua** • Warsamson valley, E of Sorong, [ca. 0°49'S, 131°24'E], 50 m, 3 Jun 1968, *Vink 17577* (BO [BO-1350055]).

Papua New Guinea. **Milne Bay** • Baniara subdistrict, W of Opanabu village, approx. 10°01'S, 149°42'E, 700 m, 10 Jul 1969, *Kanis 1195* (LAE [209030]) • Raba Raba subdist., Mayu camp I, junction of Mayu & Ugat Rives, Mt. Suckling., 9°37'S, 149°10'E, 860 m, 7 Jun 1972, *Leach & Katik LAE 56116* (BO [BO-13444471], BRI [AQ0351361], L [L.1757509], LAE [201453], SING) • ibid., 330 m, 23 Jun 1972, Leach LAE 56069 (BRI [AQ0351336], L [L.1757533]) • Maiyu River, ca. 16 km WNW of Biniguni airstrip, 9°40'S, 149°10'E, 150 m, 1 Jul 1972, *Pullen 8396* (A [A02263509], BO [BO-1372536], L [L.1757542]); **Morobe** • Quembung vicinity, 760 m, 12 Sep 1935, *Clemens 1200* (L [L.1757555]) • Kamiali Wildlife Management Area, banks of Saia River near Hessen Bay, near 7°21.7'S, 147°08.3'E, sea level, 14 Jan 2001, *Takeuchi 14912* (A [A02263510], BRIT [BRIT619081], K [K003818156], L [L.1757471], LAE [277895]) • Alluvial forest along Tabare (Tabali) River, near 7°16'S, 147°05.5'E, 75–100 m, 12–18 Jul 2001, *Takeuchi 15388* (A [A02263514], BRIT [BRIT619081], K [K003818148], LAE [286747]); **Southern Highlands** • Mt. Bosavi, Northern side, near mission station, 6°26'S, 142°50'E, 700–800 m, 6 Oct 1973, *Jacobs 9049* (L [L.1757475]) • ibid., 600–700 m, 29 Oct 1973, *Jacobs 9505* (L [L.1757478]) • Kutubu district, swamp forest adjacent to Waro and Ubogo villages, NE towards the low ridge, 6°32'S, 143°12'E, 425 m, 14 Sep 1993, *Takeuchi 9235* (A [A00935245], E [E00670271], L [L.1757519, L.1757520], US [US3316892]).

#### ﻿6. Pyramidanthe parvifolia

Taxon classificationPlantaeMagnolialesAnnonaceae

Ezedin & J.C.Lopes sp. nov.

1EE3E7D0-D7AC-52C6-B6C4-5125968DEC48

urn:lsid:ipni.org:names:77369122-1

Fig. 3D–G

##### Diagnosis.

Differs from *P. schlechteri* in bearing smaller leaves up to 5 × 2.5 cm (vs. 6–15.5 × 3–5 cm), thinner flowering pedicels, outer petals that are smaller measuring 10–12 × 4–5 mm (vs. 15–35 × 6–10 mm), and new leaves which flush out with a bronze abaxial sheen.

##### Type.

Papua New Guinea. **Sandaun** • North of junction of Bielga and Mogofola Rivers, Telefomin subdistrict, 2100 m,5°00'S, 141°05'E, 1 Jun 1975, *Vinas & Wiakabu LAE 59640* (holotype: A! [A02263537]; isotypes: BRI! [AQ0350359], L! [L.1757516], LAE! [224447]).

##### Description.

Woody climbers; twigs rusty sericeous and early glabrescent. Leaves chartaceous; petioles 5–8 mm long, terete, sharply grooved, densely rusty sericeous and very early glabrescent; laminas (2.5–)3.5–5 × (1.5–)2–2.5 cm, elliptic, adaxial surface sparsely sericeous and early glabrescent, dark green in vivo and greyish brown in sicco, abaxial surface rusty sericeous to glabrescent, brownish green in vivo and brown in sicco, base obtuse to acute, apex bluntly rounded to acute; venation eucamptodromous, primary vein sharply impressed above and raised below, secondary veins in 6–8 pairs, spaced 8–10 mm apart, angled at ca. 60° to the midvein, intersecondaries sometimes present but faint, tertiary veins irregularly percurrent to subreticulate, inconspicuous. Inflorescences axillary, 1-flowered, pedicels ca. 10 mm long, thin and wiry, ca. 1 mm wide; bracts 2, near base of the pedicel, triangular-ovate, caducous. Flowers rusty brown at anthesis; sepals 2 × 1 mm, partially connate at base, triangular ovate, slightly gibbous, outside sparsely rusty sericeous; outer petals 10–12 × 4–5 mm, narrowly triangular-ovate, subcoriaceous, outside sparsely rusty sericeous, inside densely greyish-brown tomentose; inner petals 3 × 2 mm, triangular-ovate, ca. 1 mm thick, outside mostly glabrous but densely sericeous at the apex, inside mostly glabrous but densely golden tomentose at the apex; stamens several, ca. 1.5 mm long; carpels ca. 8, ca. 1.5 mm long, ovaries ca. 0.8–1 mm long. Fruits consisting of ca. 2–3(–5) monocarps; pedicels 20 mm long, sepals caducous; stipes 3–4 mm long; monocarps ca. 10–11 × 10 mm, (irregularly) globose, the surface verruculose, glabrous, orange when ripe. Seeds not observed.

##### Etymology.

After its small leaves.

##### Distribution

**(Fig. 7).** Indonesian New Guinea and Papua New Guinea.

##### Ecology and habitat.

Found in montane forests, including degraded secondary forests, from 1060–2100 m. On slopes and ridges dominated by *Agathis* and likely Fagaceae.

##### Preliminary IUCN conservation status.

Data Deficient (DD). Only known from two localities widely spaced apart. Due to this, the EOO could not be calculated and thus, no meaningful assessment can be made. It seems likely to be found in other localities along the Central Cordillera.

##### Notes.

Currently known only from two collections that are separated by over ca. 550 km apart in distance and also by over 1000 m in altitude. Despite this distance, they are both united by their similar morphology. A couple of collections of *P. schlechteri* may bear leaves that approach those of *P. parvifolia*. Still, the latter can be distinguished by its much smaller flowers and the new leaves which display a distinct bronze abaxial sheen due to the densely appressed hairs.

The single monocarp on the type sheet at A has what appears to be an undeveloped seed, thus seed morphology could not be properly observed.

##### Additional specimens examined.

Indonesia. **West Papua** • Waponga drilling camp, 3°04'48"S, 136°20'24"E, 1060 m, 13 Apr 1998, *Ridsdale 2564* (L [L.1754996]).

#### ﻿7. Pyramidanthe schlechteri

Taxon classificationPlantaeMagnolialesAnnonaceae

(Diels) Bangk. & Chaowasku, Willdenowia 51(3): 389. 2021.

8A4FB2D4-D536-53E9-9143-BFC95694FEE3

Fig. 3D–L

Mitrella schlechteri Diels, Bot. Jahrb. Syst. 49(1): 150. 1912 ≡ Fissistigma schlechteri (Diels) Merr., Philipp. J. Sci. 15: 136. 1919. Type: [Papua New Guinea], in den Wäldern des Kani Gebirges, 1000 m, 23 December 1907, *Schlechter 17025* (holotype: B! [B 10 0325314]; isotype: P! [P00601060]).
Pyramidanthe silvatica (DIels) Bangk. & Chaowasku, Willdenowia 51(3): 389. 2021, syn. nov. ≡ Mitrella silvatica Diels, Bot. Jahrb. Syst. 52: 183. 1915. Type: [Papua New Guinea], [East Sepik], Etappenberg, 850 m, 6 Oct 1912, *Ledermann 9058* (lectotype, designated by Turner 2018, p. 577: B! [B 10 0325311]; isolectotypes: E! [E00016889], K! [K000574730], SING! [SING0045996]).

##### Type.

Based on *Mitrella schlechteri* Diels.

##### Description.

Woody climbers; twigs usually (densely) sericeous, often early glabrescent. Leaves chartaceous to (sub)coriaceous; petioles 6–13 mm long, terete, deeply grooved, densely pubescent to glabrescent; laminas 6–15.5 × 3–5 cm, variously narrowly elliptic to oblanceolate to narrowly obovate to narrowly oblong, adaxial surface glabrous to (sparsely) sericeous, mid-green in vivo and (dark) grayish-brown in sicco, abaxial surface sparsely to densely sericeous to glabrescent, glaucescent to pale rusty in vivo and orangish-brown in sicco, base variously acute to obtuse (to rounded), apex variously acuminate to acute (rarely obtuse to rounded); venation eucamptodromous (to brochidodromous), primary vein impressed above and raised below, secondary veins in 6–11 pairs, spaced 8–16(–19) mm apart, angled 55–70° to the midvein, intersecondaries frequent, tertiary veins irregularly percurrent to subreticulate, often bifacially inconspicuous. Inflorescences axillary, 1-flowered, pedicels 9–19 mm long; bracts 2–3, near the base of the pedicel, (narrowly) triangular-ovate, often persistent. Flowers creamy to yellowish to orange at anthesis; sepals 2–4 × 3–4 mm, connate at the base, broadly triangular-ovate, slightly gibbous, outside sericeous to glabrescent; outer petals 15–35 × 6–10 mm, narrowly triangular-ovate, (sub)coriaceous, outside densely dark rusty sericeous, inside grey tomentose; inner petals 5–7.5 × 1.5–3 mm, broadly triangular-ovate, outside glabrous, inside mostly glabrous except the apex with a small tuft of short hairs; stamens several, 1.5–2 mm long; carpels several, 1.5–3 mm long. Fruits consisting of 3–15 monocarps; pedicels (10–)15–25 mm long, sepals persistent; stipes 6–12 mm long; monocarps 7–14(–20) × 8–10 mm, (irregularly) globose to oblongoid, glabrous, yellow-orange to orange-brown and brown when ripe. Seeds (1–)3–5 per monocarp, light to dark reddish brown, 6–10 × 2–4 mm, subglobose to oblate or hemi-ellipsoidal, without rudimentary aril, testa smooth.

##### Distribution

**Figure 8.**
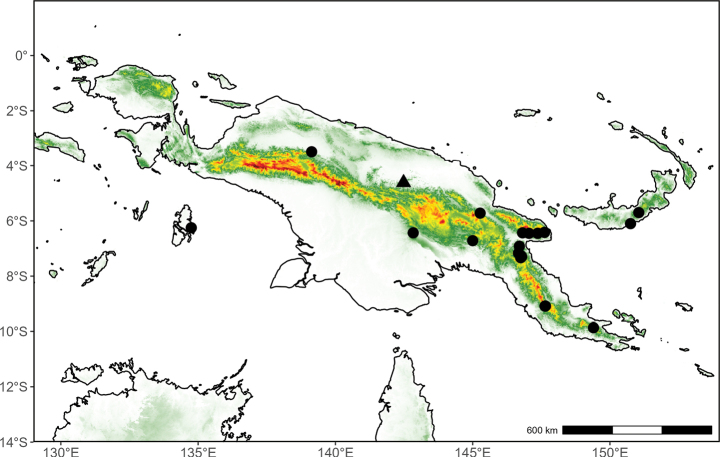
Distribution map of *Pyramidanthe schlechteri* (circle). The estimated location of the type collection of *P. silvatica* (triangle) is indicated for reference.

**(Fig. 8).** Indonesian New Guinea (Aru Is.) and Papua New Guinea.

##### Ecology and habitat.

Mostly occurring in mid-montane forests on mainland New Guinea, from (750–)1200–1600(–1900) m. On the outlying islands of Aru and New Britain found in lowland forests, from near sea level up to ca. 400 m. Found in both primary and secondary forests, including on limestone.

##### Vernacular names.

Blum (Oksapmin), Mefugo (Waskuk), Pai (Wagu).

##### Preliminary IUCN conservation status.

Least Concern (LC). The species has a very broad EOO of over 680,000 km^2^, covering a large swath of New Guinea, and is also present on Aru and New Britain, effectively shielding the species from threat.

##### Notes.

In his treatment of Papuasian Annonaceae, Diels (1915) described two new *Pyramidanthe*, namely *P. ledermannii* and *P. silvatica*. Both of these were more aligned with *P. schlechteri* than *P. beccarii*, primarily on account of the absence of a rusty indument. The type of *P. ledermannii* has broad, rounded leaves in comparison to *P. schlechteri* and *P. silvatica* whereas *P. silvatica* was differentiated from *P. ledermannii* by its narrower and more pointed outer petals and from *P. schlechteri* by the hairy ovary and by the non-thickened connective process (Diels 1915). After examining the material collected during the last century, we noticed a broad gradation in morphology of the leaves, flowers, and fruits. Yet whereas *P. ledermannii* maintains a distinct morphological unit, the distinction between *P. schlechteri* and *P. silvatica* was less clear. Thus, we synonymize *P. silvatica* under this species and adopt a broader concept of *P. schlechteri*. For characters distinguishing this species from *P. ledermannii*, see notes under that species.

Due to the variation seen in this group as currently delimited, it may likely represent a species complex. Leaves are often seen with simple appressed hairs which may sometimes be found bifacially (e.g., *Schodde 5545*). The monocarps also may vary from small globose at ca. 8 × 8 mm and single-seeded (e.g., *Henty et al. NGF 41578*) to large irregularly ovoid at ca. 20 × 10 mm (e.g., *s.n. NGF 3059*); however, there are specimens displaying both these types of monocarps (e.g., *Kairo NGF 21116*). The specimen *Harley 11488* may be confused for *P. oblongifolia* due to similar leaves, but the floral characters are completely unlike the latter with outer petals that are narrower and densely rusty tomentose. Two notable deviant subgroups can be loosely united by a couple of somewhat consistent traits: leaves narrower and monocarps small globose (*Clemens 3850*, *Foreman & Vinas 60057*, *Takeuchi 12452*, *Henty et al. NGF 41578*) and leaves thinner chartaceous and monocarps constricted (*Clemens 41797*).

##### Additional specimens examined.

Indonesia. **Maluku** • [Aru Islands], P. Kobroor, Kp. Kobroor, 6°15'S, 134°45'E, 10 m, 24 Apr 1993, v*an Balgooy & Mamesah 6469* (A [A02263530], BO [BO-0013848], L [L.1757521]).

Papua New Guinea. **Central** • Boridi, [ca. 9°05'S, 147°39'E], 1219 m, 1 Oct 1935, *Carr 14328* (SING [3 sheets]) • Above Boridi Village, Port Moresby subdistrict, 9°05'S, 147°38'E, 1280 m, 28 Sep 1973, *Foreman & Vinas LAE 60057* (A [A02263523], BRI [AQ0350362], L [L.1757553], LAE [210841]); **Eastern Highlands** • Crater Mt. Wildlife Management Area, ridge above Hauneäbäbo, ca. 6°43'S, 145°00'E, 1585–1768 m, 22 Jul 1998, *Takeuchi 12452* (A [A02263516], L [L.3727985, L.1757472]); **East New Britain** • Torlu river, Gasmat subdistrict, [ca. 5°42'S, 151°03'E], ca. 1524 m, 24 Mar 1965, *Sayers NGF 24189* (A [A02263520], BO [BO-1372539], LAE [86766, 2 sheets]); **Madang** • Numba, 5°43'19.9"S, 145°16'17"E, 1200 m, 24 Jul 2013, *Weiblen & Binatang Research Center NP2A0240* (MIN [970231]); **Milne Bay** • Mt. Simpson Range, ridge between Agaun and Bonenau, [ca. 9°52'S, 149°24'E], 1070 m, 2 Aug 1969, *Schodde 5545* (A [A02263518, A02263519], L [L.1757505], LAE [207757, 2 sheets]); **Morobe** • Partep [=Patep], [ca. 6°55'S, 146°42'E], [ca. 1550 m], [no date], *s. coll. NGF 3059* (A [A02263535) • Yunzaing, [ca. 6°25'S, 147°37'E], 1369 m, 8 Jul 1936, *Clemens 3585* (A [A02263533]) • ibid., [ca. 6°25'S, 147°37'E], 1369 m, 12 Aug 1936, *Clemens 3850* (A [A02263527]) • Ogeranang, [ca. 6°27'S, 147°22'E], 1767 m, 20 Jan 1937, *Clemens 5101* (A [A02263521]) • ibid., [ca. 6°27'S, 147°22'E], 1828 m, 19 Feb 1937, *Clemens 5448* (A [A02263528]) • Boana, [ca. 6°26'S, 146°49'E], 914–1219 m, 9 Jul 1938, *Clemens 8413A* (A [A00274959]) • Samanzing, [ca. 6°27'S, 147°03'E], 1676 m, 27 Oct 1938, *Clemens 9136* (A [A02263522]) • Boana, [ca. 6°26'S, 146°49'E], 762–1371 m, May–Nov 1940, *Clemens 41797* (A [A02263540]) • Kauli Creek, about 5 miles S of Wau, [ca. 7°22'S, 146°43'E], 1280 m, 27 Mar 1963, *Hartley 11488* (A [A02263531], BRI [AQ0210781], LAE [66615], SING) • Wau, 7°20'S, 146°45'E, 1371 m, 10 Feb 1965, *Sayers NGF 21687* (A [A02263526], BO [BO-1408117], L [L.1757554], LAE [70394, 2 sheets], SING) • Taun Creek, Bulolo, 7°10'S, 146°40'E, 914 m, 25 Jul 1965, *Kairo NGF 21116* (A [A02606779], BO [BO-1372535, BO-1816994], L [L.1757543], LAE [86025], NSW [NSW453636]) • 10.5 km NE of Wau, 7°18'S, 146°47'E, 1550 m, 12 Jan 1980, *Pratt 80-1017* (LAE [238944]); **Sandaun** • Oksapmin, Telefomin subdist., 5°20'S, 142°15'E, 1890 m, 16 Oct 1968, *Henty et al. NGF 41578* (A [A02263536], BO [BO-1372533], BRI [AQ0211103], L [L.1757546], LAE [107314]); **Southern Highlands** • Mt. Bosavi, northern side, 6°26'S, 142°50'E, 1350–1550 m, 26 Sep 1973, *Jacobs 8807* (LAE [217413]); **West New Britain** • Pirilongi village, Kandrian subdist., 6°06'S, 150°45'E, 396 m, 11 Mar 1965, *Sayers NGF 21944* (BRI [AQ0211460], L [L.1757547], LAE [87407, 2 sheets]).

### ﻿Excluded species

#### ﻿ Xylopia micrantha

Taxon classificationPlantaeMagnolialesAnnonaceae

Scheff., Ann. Jard. Bot. Buitenzorg 2: 27. 1885.

D43DE143-DB26-5E60-80E4-7E6F870B4377

##### Type.

Indonesia. Papua • Monte Arfak a Putat, Oct 1872, *Beccari PP 849* (lectotype, designated by designated by Diels 1912, pg. 151: FI-B! [FI007586] [Erb. Coll. Becc. 539]; isolectotype: B! [B 10 0365074]).

##### Notes.

This species was originally described by Scheffer (1885) who placed it in the genus *Xylopia* L. After examining the type material, Diels (1912: 150) suggested it could belong to *Mitrella* as it wouldn’t fit in *Xylopia*. Just over a century later, Turner (2018) reiterated the stance that it “probably represents a species of *Mitrella*” but stopped short of making a combination. In their revision of Papuasian *Xylopia*, Johnson and Murray (2024: 246) agreed that this species does not belong to *Xylopia* but instead to *Mitrella*.

Having revised the genus in New Guinea and examined material from other parts of Malesia, we conclude that this species does not belong to *Mitrella* (=*Pyramidanthe*). At first glance, the flowers may seem to resemble *Pyramidanthe*; however, upon closer examination, it becomes clear that several of its traits fall outside the genus. These include: 1) presence of festooned brochidodromous secondaries (vs. mostly eucamptodromous), 2) distinct intersecondaries absent or inconspicuous (vs. present), 3) tertiary veins orthogonal reticulate (vs. irregularly percurrent to subreticulate), 4) secondaries and tertiaries indistinguishable from one another (vs. always distinct), 5) both secondaries and tertiaries prominently raised on abaxial surface (vs. only secondaries or neither), and 6) outer and inner petals near the same length (vs. strongly dimorphic). This all suggests that *X. micrantha* cannot be placed confidently in *Pyramidanthe*.

Based on the type material, its affinity could be narrowed down to either *Artabotrys* R.Br., *Huberantha* Chaowasku, or *Meiogyne* Miq. Members of these genera may bear prominently raised venation on the abaxial surface with festooned brochidodromous secondaries and reticulate tertiaries. Among them, *Artabotrys* appears to be the closest match based on leaves alone. However, *X. micrantha* still cannot be confidently placed as critical information is missing from both the type and protologue. Namely, the confirmation of its growth habit (not mentioned in protologue), morphology of the inflorescence (typical hooked structures of *Artabotrys* not visible on type), morphology of the mature flower (protologue describes an immature bud), and description of fruits (not available). Due to the severe lack of information, along with the poor state of the type material, we refrain from making any combinations. For now, this name should be considered unplaced.

### ﻿New record and species of *Desmos*

The genus *Desmos* is newly reported for New Guinea based on a single fruiting specimen collected from Madang Province in Papua New Guinea that had been overlooked and unidentified. The specimen was determined to represent a new species of this genus and is here described. A comparison of the new taxa against a few other species of *Desmos* is given in Table 1.

**Table 1.** Morphological comparison between *Desmos insolens* with three other similar species.

	D. insolens	D. wardianus	D. zeylanicus	D. chinensis
Distribution	New Guinea	Australia	Sri Lanka	India, S China to Sulawesi
Forest habitat	evergreen, wet	deciduous, monsoon	evergreen, wet	evergreen, wet
Habit	scrambling shrub or liana(?)	scrambling shrub	shrub to small tree	scrambling shrub to liana
Leaf shape	narrowly elliptic to narrowly oblong	lorate	lanceolate	broadly elliptic to obovate
Leaves, abaxial color (dried)	glaucescent(?), greenish brown	glaucescent, whitish green	glaucous, blue-green to greyish white	(sub)glaucous, greyish white
Leaf apex	acute to acuminate	rounded	acuminate	acuminate
Secondary veins, anastomoses at the margin	ramifying without looping	ramifying with or without looping	fading out or ramifying then looping	fading out without looping
Perpendicular intersecondaries	present, frequent	present, infrequent	present, infrequent	absent
Tertiary veins	irregular, percurrent to reticulate	reticulate, random to orthogonal	reticulate, orthogonal	percurrent, straight
Inflorescences	supra-axillary	leaf-opposed	axillary	supra-axillary
Fruiting pedicel length (mm)	55	20–30	10–20(–25)	(20–)25–45
Stipe length (mm)	8–10	7–15	5–6	(10–)12–18

#### ﻿ Desmos insolens

Taxon classificationPlantaeMagnolialesAnnonaceae

Ezedin sp. nov.

6D736C08-0429-5764-AE62-5070277E3CBD

urn:lsid:ipni.org:names:77369123-1

Fig. 9

##### Diagnosis.

Differs from *D. wardianus* in bearing supra-axillary inflorescences (vs. leaf-opposed), narrowly elliptic to narrowly oblong leaves (vs. lorate), acute to acuminate leaf apex (vs. always rounded), longer internodes measuring 20–30 cm long (vs. 5–9 mm), sepals not persistent (vs. persistent), and longer fruiting pedicels measuring ca. 55 mm (vs. 20–30 mm). Differs from the widespread *D. chinensis* in bearing conspicuous composite intersecondary veins (vs. absent) and reticulating to irregularly percurrent tertiary venation (vs. straight percurrent).

##### Type.

Papua New Guinea. **Madang** • Josephstaal District, 4°30'S, 145°02'E, 160 m, Jun 2006, *Ama & Takeuchi s.n.* (holotype: A! [A02607250, A02607251]).

##### Description.

Woody climber or scrambling shrub; twigs glabrous, smooth, lenticellate. Leaves chartaceous; petioles 5–7 mm long, terete, grooved, glabrous; laminas (9.5–)11–14(–19) × (2.5–)3.5–4(–5.5) cm, narrowly elliptic to narrowly oblong, adaxial surface glabrous, greyish green in sicco, abaxial surface very sparsely sericeous and early glabrescent, greyish light brown in sicco, base acute, with a pair of dark-colored linear marginal glands, apex acute to acuminate; venation eucamptodromous with the terminal ends sometimes reticulating before the margins, primary vein weakly impressed above and weakly raised below, glabrous, secondary veins in (11–)19–24 pairs, adaxially faint, abaxially prominent, spaced 7–10 mm apart, angled at 40–60° to the midvein, intersecondaries present, tertiary veins abaxially prominent, irregularly percurrent to orthogonal reticulate. Inflorescences supra-axillary. Flowers unknown. Fruits consisting of ca. 15–25 monocarps, singular to moniliform and segmented, with up to 6 segments, each segment ellipsoid, 10–65 × 5–6 mm, with each segment measuring ca. 10 mm long, glabrous, terminal segment rostrate at apex, red when ripe; pedicels ca. 5.5 cm long, glabrous; torus ca. 10 mm wide, irregularly globose; stipes 8–10 mm long. Seed 1–6 per monocarp, with one per segment, light yellow, 8–10 × 5–7 mm, elliptic-oblong, testa smooth, perichalazal ring flat to weakly sunken, minutely rostrate at the distal end.

**Figure 9.**
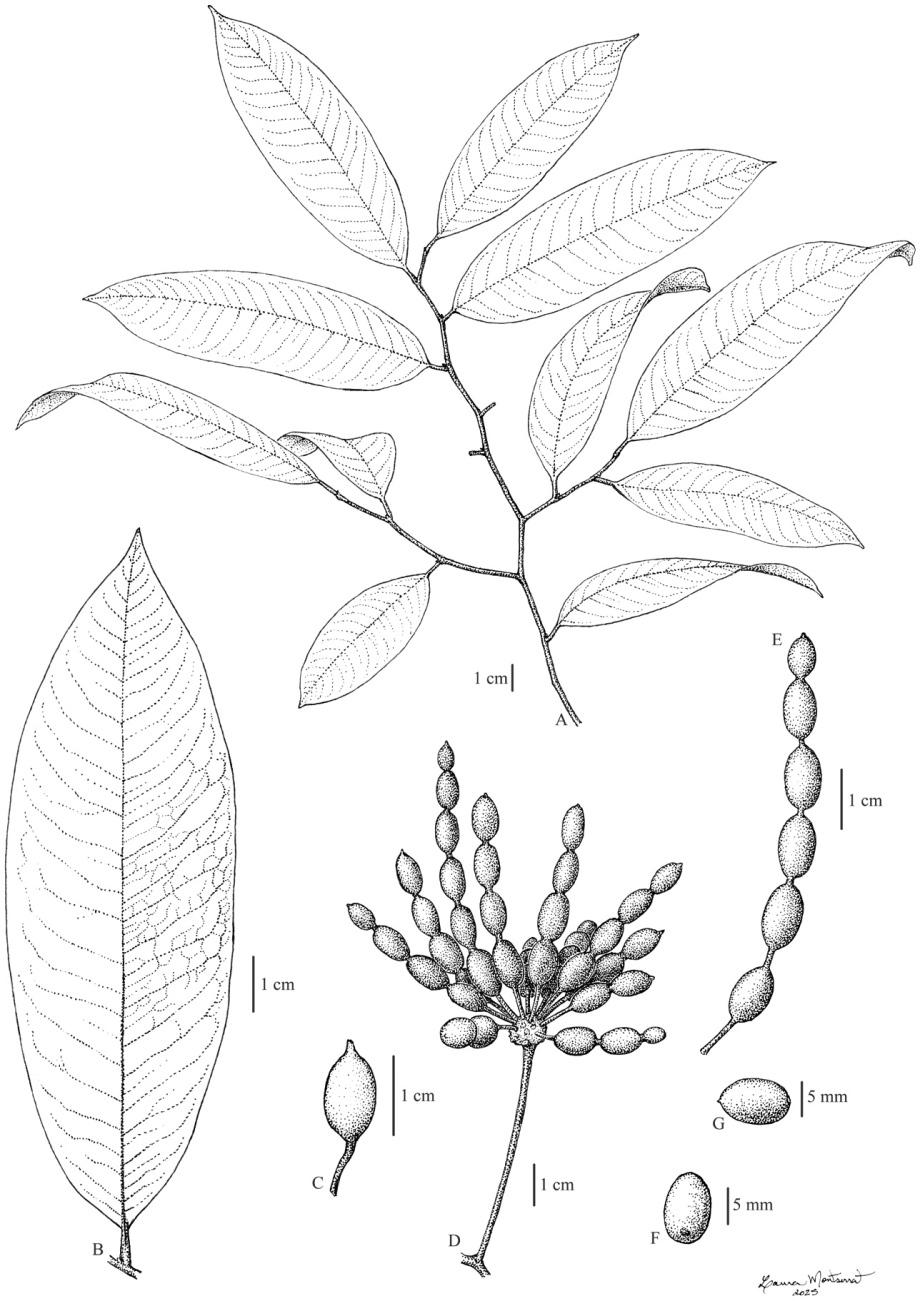
*Desmos insolens* Ezedin. A. Branch; B. Leaf with details of venation; C–E. Infructescence and individual monocarps; F, G. Seed. All from *Ama & Takeuchi s.n.* Illustrated by L. Montserrat.

##### Etymology.

From Latin *īnsolentis*, unusual; in reference to the venation.

##### Distribution

**Figure 10.**
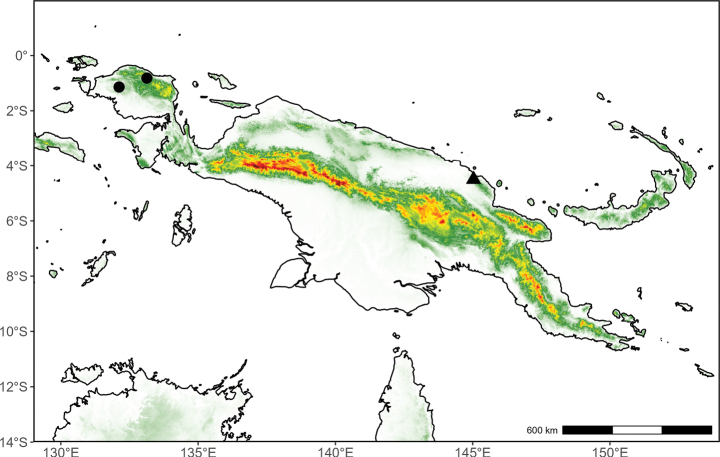
Distribution map of *Desmos insolens* (triangle) and *Fissistigma latifolium* (circle) in New Guinea.

**(Fig. 10).** Endemic to Papua New Guinea.

##### Ecology and habitat.

Lowland forest at 160 m.

##### Notes.

This new species and genus record for the island is confirmed through a single fruiting specimen comprising two sheets at A with no known duplicates or other collections. It is easily differentiated from other members of the genus by its venation, which is the most distinct feature of this species as currently known. The venation of the type collection departs quite notably from the norm in *Desmos* and the closely related *Dasymaschalon* (Hook.f. & Thomson) Dalla Torre & Harms. Leaves of both genera typically bear the same straight percurrent pattern; however, in this species, both the secondary and tertiary venation patterns are irregular and can be difficult to discern.

The secondaries are mostly eucamptodromous but sometimes can be seen reticulating, then fading out towards the margins. The tertiaries exhibit an irregularly percurrent to reticulating pattern, often with clear intersecondaries. This is unlike the widespread *D. chinensis* Lour. and most other *Desmos* as they exhibit more or less regular eucamptodromous secondaries and strait percurrent tertiaries, without prominent intersecondaries. There are only a handful of *Desmos* species that appear to depart from this norm: the Sri Lankan endemics, *D. elegans* (Thwaites) Saff. and *D. zeylanicus* (Hook.f. & Thomson) Saff., along with the Australian *D. wardianus* Jessup. The latter two exhibit a similar venation pattern to *D. insolens*, albeit with differing pedicel lengths. In particular, the Sri Lankan specimen *Kostermans 28404* (L!; as *D. zeylanicus*) bears a striking resemblance to the type specimen of *D. insolens*.

Both *Desmos* and *Dasymaschalon* are morphologically very similar and easily confused without flowering material. They also happen to be phylogenetically sister to one another (Nge et al. 2024). In the presence of flowers, they can be easily differentiated as *Desmos* bears the characteristic two whorls of petals, whereas *Dasymaschalon* bears only one whorl (Wang et al. 2012). Here, the type specimen bears fruits and no flowers. Despite the lack of flowers, the new species can be placed in *Desmos* on the basis of morphology and distribution.

The inflorescence placement in *Dasymaschalon* is known to always be axillary or terminal, whereas *Desmos* is known to vary between axillary, terminal, supra-axillary, or leaf-opposed (Kessler 1993; Guo et al. 2017b). Given the supra-axillary infructescence placement of this new species, along with the presence of marginal glands at the leaf base, we can more comfortably place it in the latter genus. Furthermore, the young twigs of *D. insolens* display numerous lenticels, which are often seen in species of *Desmos.* Whereas young twigs of *Dasymaschalon* are almost always without lenticels, although older twigs may sometimes have them. The leaves of the new species may superficially resemble those of *Dasymaschalon clusiflorum* (Merr.) Merr. but this can easily be distinguished by its axillary inflorescences, shorter fruiting pedicels (ca. 1–2 cm), lack of multi-segmented monocarps, and lack of distinct intersecondaries.

Its placement in *Desmos* is additionally supported by the distribution of the genus. *Dasymaschalon* is currently restricted to western Malesia, not extending east of Wallace’s Line. Meanwhile, *Desmos* broadly covers all of tropical Asia, reaching as far south as northern Australia, where at least three species are known to occur (Jessup 2007). Coincidentally, New Guinea just so happens to represent the only major gap in the distribution of *Desmos* as it appears to mysteriously ‘skip’ over the island (POWO 2025). With the description of *D. insolens*, a major gap in the genus’ near-continuous distribution from India to Australia can now be filled.

The specimen label notes its habit as “vine?”, bringing into question its exact habit. Although it can likely be assumed to be of some scandent nature, additional collections would help to confirm whether it is a liana or more of a scrambling shrub.

##### Conservation status.

Data Deficient (DD). Only known from its type collection. It is not known how widespread or rare this species is or whether there are other species of *Desmos* on the island. However, given its collection locality at Josephstaal, it could be assumed the species is at least present in other lowland areas of the Ramu basin or adjacent basins.

### ﻿New record of *Fissistigma* and neotypification of *F. latifolium*

The genus *Fissistigma* is newly reported for New Guinea based on two collections of *F. latifolium* from the Vogelkop Peninsula in West Papua (Indonesia). They appear to have been either overlooked or misidentified, with the earliest of the two having been collected over 60 years ago. Additionally, the species is neotypified here. The account here only deals with the two New Guinea gatherings.

#### ﻿ Fissistigma latifolium

Taxon classificationPlantaeMagnolialesAnnonaceae

(Dunal) Merr., Philipp. J. Sci. 15: 132. 1919.

C977A340-B4DF-5B7B-9C7A-D7D17D911FA7

Unona latifolia Dunal, Monogr. Anonac.: 115. 1817 ≡ Uvaria latifolia (Dunal) Blume in Fl. Javae Annon., 37 t. 15. 1830 ≡ Melodorum latifolium (Dunal) Hook.f. & Thomson, Fl. Ind. 1: 117. 1855.

##### Type.

Indonesia. Java • [no locality], [no date], *Blume s.n.* (neotype, designated here: K! [K000574675]; possible isoneotype: LE! [LE00012433]).

##### Distribution

**(Fig. 10).** Indonesian New Guinea (West Papua). Outside of New Guinea, occurring from southern China to the Moluccas.

##### Ecology and habitat.

In New Guinea, thus far known from lowland hill forests around 450 m.

##### Notes.

The type of this species was originally designated as the following description: “*Cananga sylvestris III latifolia*” in Rumphius (1741: 198) by Dunal (1817). Blume (1830) then redescribed the species as *Uvaria latifolia* with a proper Latin description and illustration based on unspecified material collected from Java, without designating a physical specimen as the type. This brief statement was again repeated by Turner (2018), who cited it as the “type” of *F. latifolium*. However, a line of text cannot serve as a type of a species since only physical specimens (gatherings) or illustrations can serve as valid type material (ICN Art. 8.1, 9.1; Turland et al. 2018).

This species has long been interpreted from a full color illustration by Blume (1830: t. XV), which was based on material that he collected during his time in Java. We know this as it was explicitly stated by Merrill (1919: 133): “the species is currently interpreted from Blume’s figure”. In the *Flora Indica* account of Hooker and Thomson (1855), the specimen “*Wall. Cat. 9411*” is listed under *Melodorum latifolium*. This notation indicates the “Wallich Catalogue” number, associated with the herbarium of the East India Company (E.I.C.) and the Herbarium Hookerianum, eventually incorporated into K in 1867. Yet searches for specimens bearing this number were unsuccessful.

Following a search on JSTOR, two Blume specimens from Java were found deposited at K and LE. Upon comparison with Blume’s illustration, it appears the illustration was most likely based on these collections due to a similar composition of the leaves and flowers. Rather than designate the illustration as the neotype, we choose to designate the original Javan material collected by Blume held at K determined as *Uvaria latifolia* as the neotype. The sheet at LE looks like it may be a possible isoneotype, however we have no definitive proof of this. The sheet at K bears the “Herbarium Hookerianum 1867” seal whereas the label of the LE specimen has the handwritten note “Herb. Blume”.

*Fissistigma latifolium* is the most widespread and variable member of its genus, ranging from S China to W New Guinea. The West Papuan collections present distinctly obovate laminas with rounded apices and cream-colored flowers, matching other collections of *F. latifolium*, including the neotype and its illustration. Collections from nearby islands of the Moluccas and Sulawesi, namely *Burley et al. 4356* (L!) and *Burley et al. 4167* (L!), also resemble the Papuan collections. It is possible that the species is more widespread in West Papua than currently known. However, it is difficult to say at this point whether or not this genus makes it into Papua New Guinea.

One collection cited here (*Vink BW 11372*) was misidentified as *Friesodielsia subaequalis* (Scheff.) R.M.K.Saunders, X.Guo & C.C.Tang. Indeed, the vegetative morphology of this species may be easily confused with that of *F. subaequalis*, which bears a striking resemblance and may even be considered nearly identical. These similarities include the densely rusty tomentose twigs and leaves, obovate laminas, conspicuous scalariform tertiaries, and an abaxial surface that is often blue-green glaucous (Ezedin 2024). One minor difference being the abaxial surface of *F. latifolium* leaves may sometimes present as glaucous, and other times not. This would likely pose an issue when trying to distinguish the two in the field without fertile material. Otherwise, *F. latifolium* may be easily differentiated from the latter by its multi-flowered inflorescences (vs. single-flowered), thick petals (vs. thin), and multi-seeded monocarps (vs. single-seeded).

See Turner (2018) for a complete list of heterotypic synonyms and their respective types.

##### Specimens examined.

Indonesia. **West Papua** • Asiti, Kebar valley, 445 m, 0°49'S, 133°08'E, 18 Feb 1961, *Vink BW 11372* (L [L.4266419], LAE [44567]) • Surroundings of Ayawasi, 1°08'24"S, 132°07'12"E, 450 m, 16 Mar 1996, *Ridsdale 2312* (A [A02263508], K [K001625851], L [L.1754957, L.1754958]).

## ﻿Discussion

The seven species of *Pyramidanthe* enumerated herein better represent the diversity found in the region as currently known. Recognition of species concepts in the genus can be somewhat tricky due to there being few consistent, stable characters, resulting in species that appear to intergrade. In general, the Papuan species can be roughly divided into three groups: flowers with subglabrous verrucose outer petals and glabrous leaves (*P. oblongifolia*), flowers with densely hairy outer petals and rusty tomentose leaves (*P. beccarii*, *P. montana*, *P. nervosa*: the ‘beccarii’ group), and flowers with densely hairy outer petals and sericeous to subglabrous leaves (*P. ledermannii*, *P. parvifolia*, *P. schlechteri*: the ‘schlechteri’ group). Although species limits within the groups may, at times, appear to occupy a morphological gradient, they can all nonetheless be distinguished from one another through the combination of multiple traits.

It is likely that this variability may be an issue found throughout the genus and not just in New Guinea. Specimens of *Pyramidanthe* that were seen from other parts of Malesia tended to similarly exhibit some level of morphological plasticity across their range, often leading species to resemble one another, particularly when sterile. Additional work is still needed to improve the taxonomy of the group in other parts of Malesia. Within New Guinea, further investigation of *P. schlechteri* is needed as the concept adopted here may likely prove to be a complex.

There does appear to be somewhat of an altitudinal signal consistent with the morphological concepts in this account. Four of the species delimited here can be considered lowland taxa as they occur in forests entirely below 1000 m (*P. beccarii*, *P. ledermannii*, *P. nervosa*, *P. oblongifolia*). The remaining three are largely restricted to the highlands above 1000 m (*P. montana*, *P. parvifolia*, and *P. schlechteri*). This pattern is not surprising since altitude often plays a significant role in determining species limits in the varied topographic landscape of New Guinea (e.g., Paul et al. 2024).

The most morphologically distinct of all the Papuan species is *P. oblongifolia*, found widespread across the lowland forests of New Guinea. Although described here as new, the oldest collection of *P. oblongifolia* dates back to 1901 – more than 120 years ago. It joins a growing list of widespread and/or common species that have been described in recent years from older collections: *Cyrtandra vittata* Bramley & H.J.Atkins (Atkins et al. 2019), *Friesodielsia papuana* Ezedin (Ezedin 2024), *Monoon excelsum* Ezedin (Ezedin 2025), *Monoon pachypetalum* I.M.Turner & Utteridge (Turner and Utteridge 2017), *Pittosporum schoddei* L.W.Cayzer & Utteridge (Cayzer et al. 2023), *Polyosma infernaralis* B.J.Conn & O.K.Paul, *P. leptorhachis* Schulze-Menz ex B.J.Conn & O.K.Paul, *P. pachyrhachis* Schulze-Menz ex B.J.Conn & O.K.Paul, *P. scyphocalyx* Schulze-Menz ex B.J.Conn & O.K.Paul (Paul et al. 2024), *Xylopia aenea* D.M.Johnson & N.A.Murray, *X. corrugata* D.M.Johnson & N.A.Murray, *X. musella* D.M.Johnson & N.A.Murray, *X. pachysericea* D.M.Johnson & N.A.Murray, and *X. rogstadii* D.M.Johnson & N.A.Murray (Johnson and Murray 2023).

This study extends the range of *Pyramidanthe* as far east as New Britain, with *P. schlechteri* occurring there. Altitudinally, it is among the highest occurring Annonaceae genera in New Guinea with *P. parvifolia* found in montane forests up to 2100 m. Current records do not show this genus occurring further eastward on other islands of the Bismarck, Louisiade, and Solomon Archipelagos. Yet this may simply be an artifact of the overall poor collecting history in these regions. This is even more of a concern for lianescent taxa which are often less commonly documented, collected, and studied in the field. Additional collections not only would contribute to our understanding of species ranges but also patterns of morphological variation.

The discovery of two genera previously unreported from New Guinea further affirms the need for renewed collections-based research on the island. Indeed, there may be similar cases where other Malesian species or even entire genera could have gone unreported and/or uncollected from the island. This is particularly relevant for the Indonesian side, which has far fewer collections yet greater proximity to the Moluccas increasing the chances of other eastern Malesian taxa being found in West Papua. The new taxa described in this work and in other recently published taxonomic treatments (e.g., Cayzer et al. 2023; Johnson and Murray 2023; Paul et al. 2024) highlight the importance of both the careful study of herbarium specimens and collecting efforts aimed at increasing our knowledge of the island’s floristic diversity.

## Supplementary Material

XML Treatment for Pyramidanthe beccarii

XML Treatment for Pyramidanthe ledermannii

XML Treatment for Pyramidanthe montana

XML Treatment for Pyramidanthe nervosa

XML Treatment for Pyramidanthe oblongifolia

XML Treatment for Pyramidanthe parvifolia

XML Treatment for Pyramidanthe schlechteri

XML Treatment for Xylopia micrantha

XML Treatment for Desmos insolens

XML Treatment for Fissistigma latifolium

## ﻿Additional information

### Use of AI

No use of AI was reported.

### Author ORCIDs

Jenifer C. Lopes https://orcid.org/0000-0001-6485-9023

### Data availability

All of the data that support the findings of this study are available in the main text.

## References

[B1] Applequist WL (2024) Report of the Nomenclature Committee for Vascular Plants: 75. Taxon 73: 288–304. 10.1002/tax.13134

[B2] Atkins HJ Heatubun CD Galloway L Bramley GLC (2019) Two new species, *Cyrtandra bungahijau* and *C. vittata*, and notes on *Cyrtandra* (Gesneriaceae) from Yapen Island, Indonesia. Kew Bulletin 74: 29. 10.1007/s12225-019-9817-2

[B3] Bachman S Moat J Hill AW De La Torre J Scott B (2011) Supporting Red List threat assessments with GeoCAT: Geospatial conservation assessment tool. ZooKeys 150: 117–126. 10.3897/zookeys.150.2109 PMC3234434 22207809

[B4] Bangkomnate R Damthongdee A Baka A Aongyong K Chaowasku T (2021) *Pyramidanthe* and *Mitrella* (Annonaceae, Uvarieae) unified: Molecular phylogenetic and morphological congruence, with new combinations in *Pyramidanthe.* Willdenowia 51: 383–394. 10.3372/wi.51.51306

[B5] Blume CL (1830) Flora Javae nec non insularum adjacentium. Annonaceae. Brusells, 1–108.

[B6] Bunchalee P Johnson DM Murray NA (2022) Distinctive new *Monoon* species (Annonaceae) from Thailand, increasing diversity of a genus of ecologically important Asian trees. Thai Forest Bulletin 50(1): 9–19. 10.20531/tfb.2022.50.1.02

[B7] Cámara-Leret R Frodin DG Adema F Anderson C Appelhans MS Argent G Arias Guerrero S Ashton P Baker WJ Barfod AS Barrington D Borosova R Bramley GLC Briggs M Buerki S Cahen D Callmander MW Cheek M Chen CW Conn BJ Coode MJE Darbyshire I Dawson S Dransfield J Drinkell C Duyfjes B Ebihara A Ezedin Z Fu LF Gideon O Girmansyah D Govaerts R Fortune-Hopkins H Hassemer G Hay A Heatubun CD Hind DJN Hoch P Homot P Hovenkamp P Hughes M Jebb M Jennings L Jimbo T Kessler M Kiew R Knapp S Lamei P Lehnert M Lewis GP Linder HP Lindsay S Low YW Lucas E Mancera JP Monro AK Moore A Middleton DJ Nagamasu H Newman MF Nic Lughadha E Melo PHA Ohlsen DJ Pannell CM Parris B Pearce L Penneys DS Perrie LR Petoe P Poulsen AD Prance GT Quakenbush JP Raes N Rodda M Rogers ZS Schuiteman A Schwartsburd P Scotland RW Simmons MP Simpson DA Stevens P Sundue M Testo W Trias-Blasi A Turner I Utteridge T Walsingham L Webber BL Wei R Weiblen GD Weigend M Weston P de Wilde W Wilkie P Wilmot-Dear CM Wilson HP Wood JRI Zhang LB van Welzen PC (2020) New Guinea has the world’s richest island flora. Nature 584: 579–583. 10.1038/s41586-020-2549-5 32760001

[B8] Cayzer LW Utteridge TM Chandler GT (2023) *Pittosporum* (Pittosporaceae) in Malesia and Papuasia. Australian Systematic Botany 36(3): 206–275. 10.1071/SB22007

[B9] Chatrou LW Pirie MD Erkens RH Couvreur TLP Neubig KM Abbott JR Mols JB Maas JW Saunders RM Chase MW (2012) A new subfamilial and tribal classification of the pantropical flowering plant family Annonaceae informed by molecular phylogenetics. Botanical Journal of the Linnean Society 169(1): 5–40. 10.1111/j.1095-8339.2012.01235.x

[B10] Chayamarit K Balslev H Johnson DM Murray NA [Eds] (2022) Flora of Thailand. Vol. 16, Part 1. Forest Herbarium, Royal Forest Department, Bangkok.

[B11] Couvreur TLP Maas PJ Meinke S Johnson DM Kessler PJ (2012) Keys to the genera of Annonaceae. Botanical Journal of the Linnean Society 169: 74–83. 10.1111/j.1095-8339.2012.01230.x

[B12] Damthongdee A Chanthamrong K Promsiri S Tongsang B Jaisamut T Wiya C Sinbumroong A Chaowasku T (2024) *Orophea chalermprakiat* (Annonaceae; Malmeoideae), a new species from southern Thailand. Phytotaxa 658(3): 296–300. 10.11646/phytotaxa.658.3.8

[B13] Diels L (1912) Die Anonaceen von Papuasien. Botanische Jahrbücher für Systematik, Pflanzengeschichte und Pflanzengeographie 49: 113–167.

[B14] Diels L (1915) Neue Anonaceen von Papuasien. Botanische Jahrbücher für Systematik, Pflanzengeschichte und Pflanzengeographie 52: 177–186.

[B15] Dunal MF (1817) Monographie de la famille des Anonacées. Treuttel et Würtz, Paris. 10.5962/t.173118

[B16] Erkens RH Blanpain LM Carrascosa Jara I Runge K Verspagen N Cosiaux A Couvreur TLP (2023) Spatial distribution of Annonaceae across biomes and anthromes: Knowledge gaps in spatial and ecological data. Plants, People, Planet 5(4): 520–535. 10.1002/ppp3.10321

[B17] Ezedin Z (2024) A synopsis of the *Friesodielsia* (Annonaceae) in New Guinea. Blumea 69: 161–170. 10.3767/blumea.2024.69.02.05

[B18] Ezedin Z (2025) An update on the genus *Monoon* (Annonaceae) in the New Guinea region. Telopea 29: 91–118.

[B19] Guo X Tang CC Thomas DC Couvreur TLP Saunders RMK (2017a) A mega-phylogeny of the Annonaceae: Taxonomic placement of five enigmatic genera and support for a new tribe, Phoenicantheae. Scientific Reports 7: 7323. 10.1038/s41598-017-07252-2 PMC5544705 28779135

[B20] Guo X Hoekstra PH Tang CC Thomas DC Wieringa JJ Chatrou LW Saunders RMK (2017b) Cutting up the climbers: Evidence for extensive polyphyly in *Friesodielsia* (Annonaceae) necessitates generic realignment across the tribe Uvarieae. Taxon 66(1): 3–19. 10.12705/661.1

[B21] Hewson HJ (2019) Plant Indumentum: A Handbook of Terminology. Australian Biological Resources Study, Commonwealth Department of the Environment and Energy, Canberra.

[B22] Hickey LJ (1973) Classification of the Architecture of Dicotyledonous Leaves. American Journal of Botany 60: 17–33. 10.1002/j.1537-2197.1973.tb10192.x

[B23] Hooker JD Thomson T (1855) Flora Indica. Being a Systematic Account of the Plants of British India. 1: 86–153. [W. Pamplin, London.] 10.5962/bhl.title.50109

[B24] Jessup LW (2007) Annonaceae. Flora of Australia 2: 18–57. ABRS/CSIRO, Melbourne.

[B25] Johnson DM Murray NA (2023) A contribution to the systematics of *Xylopia* (Annonaceae) in the New Guinea region. Gardens’ Bulletin (Singapore) 75: 183–255. 10.26492/gbs75(2).2023-02

[B26] Kessler PJA (1993) Annonaceae. In: Kubitzki K Rohwer JG Bittrich V (Eds) The Families and Genera of Vascular Plants.Vol. 2. Springer Verlag, Berlin, 93–129. 10.1007/978-3-662-02899-5_9

[B27] Kessler PJA Jessup LW Kruijer JD (1995) Provisional checklist of the Asiatic-Australian species of Annonaceae. The Herbarium, Universiti Pertanian Malaysia, Serdang.

[B28] Knapp S (2024) A revision of *Lycianthes* (Solanaceae) in tropical Asia. PhytoKeys 245: 1–106. 10.3897/phytokeys.245.121988 39113755 PMC11301032

[B29] Merrill ED (1919) On the application of the generic name *Melodorum* of Loureiro. Philippine Journal of Science (Botany) 15: 125–137.

[B30] Miquel FAW (1865) Anonaceae Archipelagi indici. Ann. Mus. Bot. Lugduno-Batavi 2: 1–45.

[B31] Nge FJ Chaowasku T Damthongdee A Wiya C Soulé VR Rodrigues‐Vaz C Bruy D Mariac C Chatrou LW Chen J Choo LM Dagallier L-PMJ Erkens RHJ Johnson DM Leeratiwong C Lobão AQ Lopes JC Martínez-Velarde MF Munzinger J Murray NA Neo WL Rakotoarinivo M Ortiz-Rodriguez AE Sonké B Thomas DC Wieringa JJ Couvreur TP (2024) Complete genus‐level phylogenomics and new subtribal classification of the pantropical plant family Annonaceae. Taxon 73: 1341–1369. 10.1002/tax.13260

[B32] Page N (2023) A new species of *Meiogyne* (Annonaceae) from the Eastern Himalayas of northeast India. Edinburgh Journal of Botany 80: 1954. 10.24823/EJB.2023.1954

[B33] Paul OK Conn BJ Henwood MJ (2024) Taxonomic review of *Polyosma* (Escalloniaceae) in Papuasia. Blumea 69(1): 54–88. 10.3767/blumea.2024.69.01.07

[B34] Pelser PB, Barcelona JF, Nickrent DL [Eds] (continuously updated) Co’s Digital Flora of the Philippines. www.philippineplants.org

[B35] POWO (2025) Plants of the World Online. Facilitated by the Royal Botanic Gardens, Kew. http://www.plantsoftheworldonline.org/ [Retrieved 12 February 2025]

[B36] Randi A Wijedasa LS Thomas DC (2022) *Disepalum rawagambut* (Annonaceae), a new tree species from peat swamp forest of Sumatra, Indonesia. Phytotaxa 530(1): 121–126. 10.11646/phytotaxa.530.1.14

[B37] Rumphius GE (1741) Herbarium amboinense. Apud Franciscum Changuion, Amsterdam.

[B38] Scheffer RHCC (1885) Sur quelques plantes Nouvelles ou peu connues de l’Archipel Indien. Annales du Jardin Botanique de Buitenzorg 2: 1–31.

[B39] Soepadmo E Saw LG Chung RCK Kiew R (Eds) (2014) Tree Flora of Sabah and Sarawak. Vol. 8. Forest Research Institute Malaysia, Kuala Lumpur.

[B40] Su YCF Saunders RMK (2006) Monograph of *Pseuduvaria* (Annonaceae). Systematic Botany Monographs 79: 1–204. https://www.jstor.org/stable/i25027953

[B41] Thiers BM (continuously updated) Index Herbariorum. https://sweetgum.nybg.org/science/ih/ [accessed 12 December 2024]

[B42] Turland NJ Wiersema JH Barrie FR Greuter W Hawksworth DL Herendeen PS Knapp S Kusber W-H Li D-Z Marhold K May TW McNeill J Monro AM Prado J Price MJ Smith GF (Eds) (2018) International Code of Nomenclature for algae, fungi, and plants (Shenzhen Code) adopted by the Nineteenth International Botanical Congress Shenzhen, China, July 2017. Regnum Vegetabile 159. Koeltz Botanical Books, Glashütten. 10.12705/Code.2018

[B43] Turner IM (2011) A catalogue of the Annonaceae of Borneo. Phytotaxa 36: 1–120. 10.11646/phytotaxa.36.1.1

[B44] Turner IM (2012) Annonaceae of Borneo: A review of the climbing species. Gardens’ Bulletin (Singapore) 64: 371–479.

[B45] Turner IM (2018) Annonaceae of the Asia-Pacific region: Names, types and distributions. Gardens’ Bulletin (Singapore) 70: 409–744. 10.26492/gbs70(2).2018-11

[B46] Turner IM (2022) (2883) Proposal to conserve the name *Mitrella* against *Pyramidanthe* (Annonaceae). Taxon 71: 476–477. 10.1002/tax.12702

[B47] Turner IM Utteridge TMA (2017) Annonaceae in the Western Pacific: Geographic patterns and four new species. European Journal of Taxonomy 339: 1–44. 10.5852/ejt.2017.339

[B48] Wang J Thomas DC Su YCF Meinke S Chatrou LW Saunders RMK (2012) A plastid DNA phylogeny of *Dasymaschalon* (Annonaceae) and allied genera: Evidence for generic non-monophyly and the parallel evolutionary loss of inner petals. Taxon 61(3): 545–558. 10.1002/tax.613005

[B49] Yang B Li JY Yang RJ Ding HB Deng M Xiao CF Zuo YJ Tan YH (2023) Two new species of *Polyalthiopsis* (Annonaceae) based on morphological characters and phylogenetic evidence, with a supplementary description of *P. chinensis* from China. Plant Diversity 45(2): 185–198. 10.1016/j.pld.2022.05.001 37069923 PMC10105080

